# The effect of low childhood income on self-harm in young adulthood: Mediation by adolescent mental health, behavioural factors and school performance

**DOI:** 10.1016/j.ssmph.2021.100756

**Published:** 2021-02-18

**Authors:** Joonas Pitkänen, Maarten J. Bijlsma, Hanna Remes, Mikko Aaltonen, Pekka Martikainen

**Affiliations:** a Population Research Unit, Faculty of Social Sciences, University of Helsinki, P.O. Box 18, FIN-00014, Helsinki, Finland; b International Max Planck Research School for Population, Health and Data Science, Max Planck Institute for Demographic Research, Rostock, Germany; c Max Planck Institute for Demographic Research, Rostock, Germany; d Institute of Criminology and Legal Policy, Faculty of Social Sciences, University of Helsinki, Helsinki, Finland; e Law School. University of Eastern Finland, Joensuu, Finland; f Department of Public Health Sciences, Stockholm University, Sweden

**Keywords:** Childhood income, Self-harm, Adolescence, Young adulthood, Mediation analysis, G-formula

## Abstract

Low childhood income is an established risk factor of self-harm in adolescence and young adulthood, and childhood income is additionally associated with various correlates of self-harm. How these correlates, such as psychiatric disorders, substance abuse, violent behaviour and school problems, mediate the effect of childhood income on self-harm, is less understood. The purpose of the current paper is to examine this mediation. The study is based on administrative register data on all Finnish children born in 1990–1995. An analytical sample of 384,121 children is followed from age 8 to 22. We apply the parametric g-formula to study the effect of childhood income on the risk of self-harm in young adulthood. Adolescent psychiatric disorders, substance abuse, prior self-harm, violent criminality and victimization, out-of-home placements, not being in education, employment or training and school performance are considered as potential mediators. We control for confounding factors related to childhood family characteristics. As a hypothetical intervention, we moved those in the lowest childhood income quintile to the second-lowest quintile, which resulted in a 7% reduction in hospital-presenting self-harm in young adulthood among those targeted by the intervention (2% reduction in the total population). 67% of the effect was mediated through the chosen mediators. The results indicate that increases in childhood material resources could protect from self-harm in young adulthood. Moreover, the large proportion of mediation suggests that targeted interventions for high-risk adolescents may be beneficial. To our knowledge, this is the first paper to use the parametric g-formula to study youth self-harm. Future applications are encouraged as the method offers several further opportunities for analysing the complex life course pathways to self-harm.

## Highlights

• We study mediation in the association between low childhood income and self-harm.
• Effects of a hypothetical intervention are examined.
• Most of the effect of low childhood income on the risk of self-harm is indirect.
• Distinct pathways are identified.
• The parametric g-formula offers further avenues for self-harm research.

## 1 Introduction

The life-time prevalence of self-harm among adolescents has been estimated to be over 15% (Gillies et al., 2018; Muehlenkamp et al., 2012), and roughly 10% of all self-harm episodes lead to hospital admission (Gillies et al., 2018; Madge et al., 2008). Moreover, non-lethal self-harm is a strong predictor of subsequent suicide (Hawton et al., 2012a, 2012b), which is globally one of the leading causes of youth mortality (World Health Organization, 2019). In addition to economic costs at state level (Tsiachristas et al., 2017), self-harm has major negative consequences for the well-being of individuals (Mars et al., 2014), and their families and friends (Ferrey et al., 2016).

A known risk factor for self-harming behaviour in adolescence and young adulthood is low childhood income (Hawton et al., 2012b, Hawton et al., 2012a; Mok et al., 2018; Page et al., 2014). Low income can be considered a stressor, which may affect child socio-emotional, behavioural and cognitive development (Berger et al., 2009; Hodgkinson et al., 2017) as well as the development of coping strategies (Kim et al., 2016). These issues are reflected in the diverse motives and functions of self-harm that, besides suicidality, include, e.g., emotion regulation, coping, self-punishment and interpersonal communication (Edmondson et al., 2016). Lack of disposable resources may also affect children indirectly through limited access to health care and consequent untreated health conditions (Hodgkinson et al., 2017), and impaired parenting due to poverty-related stress in parents (Berger et al., 2009). Moreover, adverse childhood experiences, such as parental mental health or substance abuse problems and family dissolutions, are more common in low-income households (Halfon et al., 2017). These experiences are known to have an impact on adolescent mental health problems and self-harm (Björkenstam et al., 2016a, 2016b), and recent findings also suggest that the consequences of adverse experiences may be more detrimental in low-income households than in others (Lanier et al., 2018; Pitkänen, Remes, Aaltonen, & Martikainen, 2019).

In addition to self-harm, low childhood income has been associated with adolescent mental health (Fitzsimons et al., 2017), high-risk health behaviours, such as substance abuse and violent offending (Kipping et al., 2015) and with an increased risk of violent victimization (Aaltonen et al., 2012). Moreover, low childhood income is known to predict out-of-home placements (Gypen et al., 2017), poor school performance (Vinnerljung & Hjern, 2011) as well as disrupted transitions to school and employment (Pitkänen, Remes, Moustgaard, & Martikainen, 2019). Besides being harmful in themselves, all of these factors may act as pathways from low childhood income to self-harming behaviour (Dube et al., 2001; Evans et al., 2004; Hawton, Saunders, & O’Connor, 2012; Henderson et al., 2017; Jablonska et al., 2012; Kääriälä & Hiilamo, 2017; Lodebo et al., 2017; Perez et al., 2016; Vaughn et al., 2015).

Previous work on possible mediating variables between childhood socioeconomic status (SES) and youth self-harm has shown that parental education and family income are associated with self-harm after adjusting for adolescent mental health but the associations are attenuated (Hawton et al., 2012b, Hawton et al., 2012a; Page et al., 2014). School performance is an important mediator between SES and youth self-harm in Sweden (Hawton et al., 2012b, Hawton et al., 2012a). Among adults, SES is associated with suicide attempts both directly and indirectly through mental health problems, substance abuse and negative life events (Aschan et al., 2013). Moreover, aggression, impulsivity, school performance and substance abuse are shown to mediate the effects of childhood adversity on self-harm among juveniles (Perez et al., 2016), and psychopathology and school performance at the population level (Björkenstam et al., 2016b). Violent offending partly mediates the association between childhood adversity and suicide (Björkenstam et al., 2018). However, what is missing from the literature is a population-level study that examines the effect of childhood socioeconomic status on self-harm and how this effect is mediated through a multitude of adolescent pathways.

**Fig. 1:**
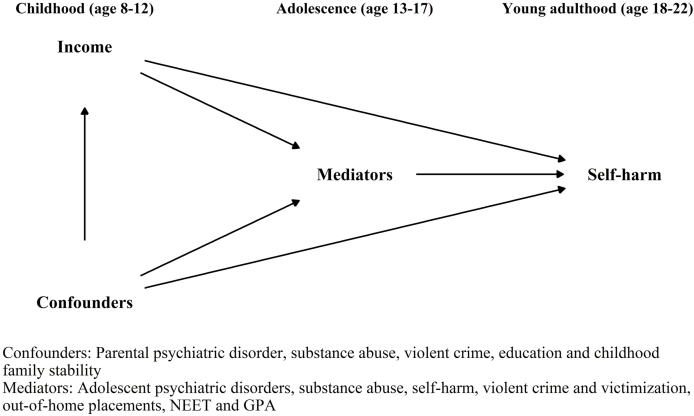
Directed Acyclic Graph of the hypothesized associations between childhood income and self-harm in young adulthood, mediated through factors and events in adolescence and confounded by several childhood factors and experiences.

To bridge this gap in research, we assess the contribution of childhood income on self-harm, adjusting for parental education and adversity, and the extent to which this effect is mediated by adolescent mental health, behavioural problems and school performance (Fig. 1). Thus, we adopt the idea of fundamental causes of disease (Link & Phelan, 1995) by conceptualizing low income as the distal factor that is associated with several proximal variables and further with self-harm. Finland provides an excellent context for this type of study with accurate linkages between annually updated censuses and other registers, such as hospital discharge and child welfare register. The prevalence of youth self-harm in Finland is similar to that found in international meta-analyses (Hawton et al., 2012b, Hawton et al., 2012a; Laukkanen et al., 2009). In terms of self-harm presenting to treatment, Finland has a comparable prevalence (1%) to that of Sweden (Sourander et al., 2009; Björkenstam et al., 2016b).

As a methodological novelty to the field of youth self-harm research, we implement the parametric g-formula. Originally developed for modelling dynamic treatment regimes, the method has several advantages in mediation analysis. The g-formula allows for modelling with generalized linear models while avoiding through standardization the non-collapsibility problems that commonly arise when comparing nested non-linear models (Vansteelandt & Keiding, 2011). With rare events, mediation analysis is possible with simpler, more traditional approaches, since the problems related to non-collapsibility diminish when outcome is sufficiently rare (VanderWeele, 2016). However, this requires that the outcome is rare within all the exposure-mediator combinations (Samoilenko & Lefebvre, 2019), making g-formula a good choice if an overall rare outcome is common in some strata.

Another candidate to avoid non-collapsibility with mediation analysis of binary outcomes would be the KHB analysis (Karlson et al., 2012). However, the g-formula is more flexible than KHB and performs equally well, or even better in certain settings (Linden & Karlson, 2013). Lastly, the g-formula allows for designing custom interventions, which is a property lacking from other methodsand the effect estimates produced are easily understandable population-averaged effects. Both of these properties can be helpful for estimating the effects of hypothetical policy interventions (Hawton et al., 2012b, Hawton et al., 2012a; Keil et al., 2014). As a trade-off, the g-formula is computationally more extensive than simpler methods are.

Our application of the g-formula introduces a hypothetical scenario of raising those in the lowest childhood income quintile into the second-lowest quintile, while holding childhood parental and family characteristics as observed. We examine how the effect of childhood income on self-harm is mediated through adolescent psychiatric disorders, substance abuse, previous self-harm, violent victimization, violent criminality, not being in education, employment or training (NEET), school performance and out-of-home placements. Our potential confounders include childhood experience of parental psychiatric disorders, substance abuse and violent criminality, as well as parental education and family stability. We also assess whether mediation effects vary by each mediator and if these effects are different in population sub-groups defined by parental characteristics. To our knowledge, this is the first study to examine at the population level how the effect of childhood income on self-harm in young adulthood is mediated through a wide array of adolescent factors, simultaneously including mental health, behavioural problems and school performance.

## 2 Methods

### 2.1 Data

The study is based on administrative register data on all the Finnish 0–14-year-old children in 2000 linked with register data on their biological parents. The dataset includes annually updated information on social and demographic indicators, causes of death (Statistics Finland, 1987–2017), hospital discharges (National Institute for Health and Welfare, 1987–2017), specialized outpatient health care service use (National Institute for Health and Welfare, 1998–2017), as well as information on child welfare (National Institute for Health and Welfare, 1991–2017) and criminal offences known to the Finnish police (1996–2017). The study has been approved by the Statistics Finland Board of Statistical Ethics (TK-53-1121-18).

To achieve symmetrical measurement periods for each variable and for each individual in the data, we limited our analyses to birth cohorts 1990–1995 (N = 392,520) and assessed childhood factors at age 8–12. We followed all the children from childhood through adolescence (age 13–17) to young adulthood (age 18–22). We excluded children with missing data due to being absent from any annual census between ages 8 and 17 (N = 4,543) and those not in the census at any point in young adulthood (N = 422). In addition, we excluded children who could not be linked to their parents (N = 836) and children missing all information on childhood family income (N = 2,598). After these exclusions (2.1%) our final sample size was 384,121 individuals.

### 2.2 Outcome

We derived information on self-harm from the hospital discharge register and the data on specialized outpatient health care service use. We used the International Classification of Diseases version 10 (ICD-10) and defined as self-harm all the episodes registered with codes X69-X84 (the Finnish classification includes all self-inflicted poisoning under code X69). This information was supplemented with all the suicides (ICD-10 X60-X84, Y870) occurring between age 18 and 22 (N = 336). Most of the self-harm cases are due to self-inflicted poisoning. Due to the use of administrative data, our measure of self-harm includes the visible part of the iceberg model of self-harm (Hawton et al., 2012b) and are likely to be severe.

### 2.3 Exposure

We measured childhood income by using information on disposable income of all family members including salaries, entrepreneurial and property income as well as income transfers. We adjusted the disposable income for inflation and divided it by household consumption units (OECD modified scale). For each individual, we calculated the mean income between age 8 and 12 and, from these mean values, quintiles were calculated.

### 2.4 Confounders

We included parental psychiatric disorders, substance abuse, violent criminality, parental education and family stability measured at age 8–12 as potential confounders. We combined information on biological parents and used binary indicators for parental psychiatric disorders, substance abuse and violent crime (ever during childhood/no experience). Family stability was measured using information on household family structure. In addition, we adjusted for parental education, year of birth (categorical) and biological sex in all our models.

Hospital discharge and specialized outpatient health care service use records were used to define parental psychiatric disorders from main and secondary diagnoses. The ICD-codes used are available in Supplementary File 1. All inpatient and outpatient episodes were included in these measures. We supplemented parental substance abuse with suspected offences of driving under the influence. Parental violent criminality was defined as any suspected violent offence and parental education as the highest level of education attained by either one of the parents (basic/upper secondary/any tertiary).

In order to capture different family transitions during adolescence, the family stability variable was defined as follows. First, we identified children living with two parents, single parents and living without parents (institutions or foster parents) at age 8–12. Second, we identified the number of changes in the family during this age period, which resulted in the following classification: intact two-parent family, intact single-parent family, multiple changes in family structure, disrupted two-parent family (from two-parent to single parent), re-partnered single parent (from single parent to two-parent family) and ever without family.

### 2.5 Mediators

As possible mediators in the association between childhood family income and self-harm, we included psychiatric disorders, substance abuse, self-harm, violent criminality and victimization, out-of-home placements, not being in education, employment or training (NEET) and school performance in adolescence (age 13–17).

We used the same strategy as with the parents to measure psychiatric disorders, substance abuse and violent criminality among the adolescents. Violent victimization in adolescence was derived from the same health care data (ICD10, Supplementary file 1). We also included a measure of self-harm in adolescence as a mediator, since history of self-harm is a strong predictor of subsequent self-harm episodes. Adolescent self-harm was measured in a similar manner as self-harm in young adulthood, excluding suicides (the analyses are restricted to children who are alive at the age of 18). We used binary indicators (ever during adolescence/no) for these measures.

NEET was defined as being unemployed or outside the labour force, but not retired or studying. The information was derived from Statistics Finland's data on main type of activity at the end of each year. A binary indicator was used in the analyses. School performance at the end of compulsory schooling was defined using information on grade point averages (GPAs) from Statistics Finland. We divided GPAs into quartiles. Since the GPA is registered only if the adolescent has applied for secondary school, usually at the age of 16, there were individuals with missing data (3%). We included these children in the lowest quartile since it is likely that – in the Finnish context where applying for secondary school is nearly universal and heavily encouraged during the last years of comprehensive school – they have a low GPA.

Lastly, all of the children that had any record of placement at age 13–17 in the child welfare register were defined as being placed out of home. In contrast to placements during childhood, out-of-home placements in adolescence are usually due to adolescent behavioural problems.

### 2.6 The parametric g-formula

We used the parametric g-formula to estimate the total and mediated effects of a hypothetical income intervention on self-harm (Bijlsma et al, 2017, 2019; Keil et al., 2014). We assess the theoretical validity of our model in supplementary file 2, and provide a general walkthrough and cleaned and cut R code of our g-formula in Supplementary files 3 and 4. Here we give a brief overview of the method.

The g-formula starts by sampling with replacement from the original data and fitting appropriate models for the mediators and outcome. We used logistic regression to model all our variables. The models for mediators included all the childhood confounders, sex and birth year. The categorical mediator GPA was modelled with a series of logistic regressions. The model for the outcome, self-harm in young adulthood, additionally included all the mediators (Odds ratios (OR) from the mediator models are available in Supplementary file 5. For the outcome, ORs are presented in Table 1 and Supplementary file 5). We evaluate the correct specification of the models in Supplementary file 6.

**Table 1:** Distributions of childhood income, mediators and confounding variables, prevalence of self-harm by category and Odds Ratios from the underlying model for self-harm at age 18–22.

Childhood income	N	%	Self-harm in young adulthood (age 18–22) %	OR	95% CI	
***Quintiles***						
Highest	76824	20.0	0.5	ref.		
4th	76824	20.0	0.7	1.10	0.96	1.25
3rd	76824	20.0	0.9	1.17	1.03	1.33
2nd	76824	20.0	1.1	1.21	1.06	1.37
Lowest	76825	20.0	1.4	1.24	1.08	1.41
**Adolescent mediators**						
*Self-harm 13–17*						
No	383087	99.7	0.9	ref.		
Yes	1034	0.3	17.4	4.64	3.85	5.56
*Psychiatric disorder 13–17*						
No	350512	91.3	0.6	ref.		
Yes	33609	8.7	4.1	3.41	3.14	3.70
*Substance abuse 13–17*						
No	379106	98.7	0.9	ref.		
Yes	5015	1.3	6.2	1.74	1.51	1.99
*Violent victimization 13–17*						
No	383106	99.7	0.9	ref.		
Yes	1015	0.3	3.7	1.29	0.90	1.81
*Violent crime 13–17*						
No	374646	97.5	0.8	ref.		
Yes	9475	2.5	4.1	1.81	1.60	2.04
*Out-of-home placement 13–17*						
No	373231	97.2	0.8	ref.		
Yes	10890	2.8	6.9	2.28	2.05	2.53
*NEET 13–17*						
No	355220	92.5	0.8	ref.		
Yes	28901	7.5	2.7	1.36	1.24	1.49
*Quartiles of GPA*						
Highest	89766	23.4	0.4	ref.		
3rd	95763	24.9	0.6	1.39	1.22	1.58
2nd	93297	24.3	1.0	1.83	1.62	2.08
Lowest	105295	27.4	1.6	2.20	1.94	2.49
**Childhood confounders**						
*Parental psychiatric disorder*						
No	352767	91.8	0.8	ref.		
Yes	31354	8.2	1.9	1.26	1.14	1.39
*Parental substance abuse*						
No	360945	94.0	0.8	ref.		
Yes	23176	6.0	2.1	1.21	1.08	1.35
*Parental violent crime*						
No	367372	95.6	0.9	ref.		
Yes	16749	4.4	2.0	1.06	0.93	1.20
*Family stability*						
Intact two-parent	282479	73.5	0.7	ref.		
Intact single-parent	41703	10.9	1.6	1.26	1.14	1.39
Multiple changes	11488	3.0	1.8	1.38	1.18	1.61
Ever out of family	4241	1.1	2.3	1.15	0.92	1.43
Disrupted two-parent	28080	7.3	1.4	1.21	1.07	1.35
Repartnered single-parent	16130	4.2	1.4	1.20	1.04	1.39
*Parental education*						
Tertiary	204239	53.2	0.7	ref.		
Secondary	157413	41.0	1.1	1.04	0.96	1.13
Basic	22469	5.8	1.7	1.04	0.92	1.18

OR: Adjusted odds ratios from the underlying model used in simulations in the g-formula. Model fit to observational data.

In total, 3,557 individuals had self-harmed at least once in young adulthood.

With these model parameters, we predicted probabilities for each mediator and the outcome. These predicted probabilities were then used to draw values from binomial distributions to produce a new dataset without intervention. We refer to this process as “simulation” from here on. This step aimed at reproducing the observed data and can thus be called the natural course scenario (NC) (Hawton et al., 2012b, Hawton et al., 2012a). As a next step, we intervened on childhood income and raised those in the lowest income quintile to the second-lowest, while leaving confounders as observed. The data with the hypothetical intervention were then used to simulate values for the mediators and the outcome. The resulting data set is called the counterfactual scenario (CF). Lastly, to assess mediation, we simulated a new scenario, where the mediator values were derived from the NC and income was intervened on in a similar manner as in the CF.

For each mediator and the outcome, the average values over the simulated scenarios were saved. These averages represent the proportion of individuals with the mediator or outcome in each scenario. To reduce Monte Carlo error, the simulations and calculations of average values were repeated 100 times and the average of the 100 Monte Carlo iterations was then used as the estimate in effect calculation. The population-averaged absolute total effect (TE) of the intervention on self-harm was defined as the difference between these stabilized estimates (averages of the mediator or outcome) in the counterfactual and natural course scenarios, and relative TE, the percentage reduction in the proportion of individuals with the mediator or outcome between the NC and CF scenarios, as one minus the ratio between these estimates. We performed the above steps 250 times to obtain 95% confidence intervals.

For ease of interpretation, we present the total effects in relative terms, i.e. as the percentage change between the counterfactual and natural course simulations in the proportion of individuals with given outcome. As the outcomes of interest are rare, population-level absolute effects are small, even if the intervention would eradicate all occurring self-harm cases. Moreover, as the prevalence of mediators differs, relative effects allow for comparison between variables. Absolute effect sizes are available in Supplementary file 7. We provide estimates of the population-averaged effects as well as average treatment effects for the treated (ATT). The latter represents the effect of income on self-harm among those who are affected by the hypothetical intervention.

Our mediation scenario corresponds to the decomposition of effects into Total Direct Effect (TDE) and Natural Indirect Effect (NIE) (Wang & Arah, 2015). The difference in the estimates between the natural course and the mediation scenario is the TDE, and NIE is derived by subtracting TDE from TE. We use percentage mediated to illustrate the magnitude of mediation (the ratio of NIE to TE). Due to small and statistically insignificant TDE (see results), which relates to the design of the intervention (modest change in income) as well as self-harm in young adulthood being a rare event, the calculation of these ratios within bootstrap iterations was unstable. Therefore, we calculated the average NIE and average TE across all the bootstrap iterations and used the ratio of these averages as the estimate of the percentage mediated. We present 95% confidence intervals for this estimate based on the normality assumption, but we found very similar results when using Fieller's theorem (Fieller, 1954). Moreover, we present density plots of all the effects in Supplementary File 8, in order to disclose the distributions of the effects across all the bootstrap iterations.

### 2.7 Additional analyses

We assessed mediation further by letting our intervention on income affect each of the mediators one at a time while holding the other mediators at natural course values, and calculated TDE and NIE from all the scenarios (Hawton et al., 2012b, Hawton et al., 2012a), thus allowing us to assess the relevance of specific mediating factors. In practice, this operation includes the effect of the chosen mediator in the total direct effect. Therefore, the lower the percentage mediated compared to the scenario with all mediators, the larger the contribution of the individual mediator for the total amount of mediation. Due to the non-linear nature of the underlying models, the individual mediation effects are not additive but provide insights into specific pathways through which low income impacts self-harm (Hawton et al., 2012b, Hawton et al., 2012a).We also examined whether the direct effect of childhood income on self-harm and indirect effect through the mediators differed in sub-populations defined by experiences of childhood adversity. We assessed such effect modification by parental psychiatric disorders, substance abuse, violent criminality and family stability.

Finally, due to the known issues related to modelling of rare events with logistic regression (King & Zeng, 2001), we did a small-scale simulation study to assess the robustness of our findings (Supplementary file 8). We show that, with the large sample size, the rareness of outcome does not influence either the effect calculations of the g-formula or underestimate the prevalence of the outcome modelled with logistic regression.

## 3 Results

### 3.1 Descriptive statistics

The prevalence of self-harm by childhood income and mediator variables is presented in Table 1. Table 1 also includes odds ratios from the underlying model used in g-formula simulations (See Supplementary File 5 for gender and birth year). In the observed data, 3,557 (0.9%) individuals had been admitted to care or died due to self-harm in young adulthood. The prevalence of self-harm in the lowest childhood income quintile was nearly three times the prevalence in the highest (1.4% vs. 0.5%) and in the fully adjusted model used in the g-formula, the odds of self-harm were around 20% greater in the lowest quintile when compared to the highest (OR = 1.24, 95% CI: 1.08, 1.41). All the mediators were positively associated with self-harm, except for violent victimization. The strongest association was between self-harm in adolescence and self-harm in young adulthood (17% vs. 0.9%, adjusted OR = 4.6, 95% CI: 3.85, 5.56). Poorer school performance predicted self-harm with a clear difference between the highest and lowest GPA quartiles (1.6% vs. 0.4%, adjusted OR = 2.2, 95% CI 1.94, 2.49). Of the confounders, experience of parental substance abuse or psychiatric disorder increased the odds of self-harm by 20% in the adjusted model, and living in an intact two-parent family seemed to protect from self-harm when compared to the other categories of the family stability variable.

### 3.2 Total effect of the hypothetical intervention

**Fig. 2:**
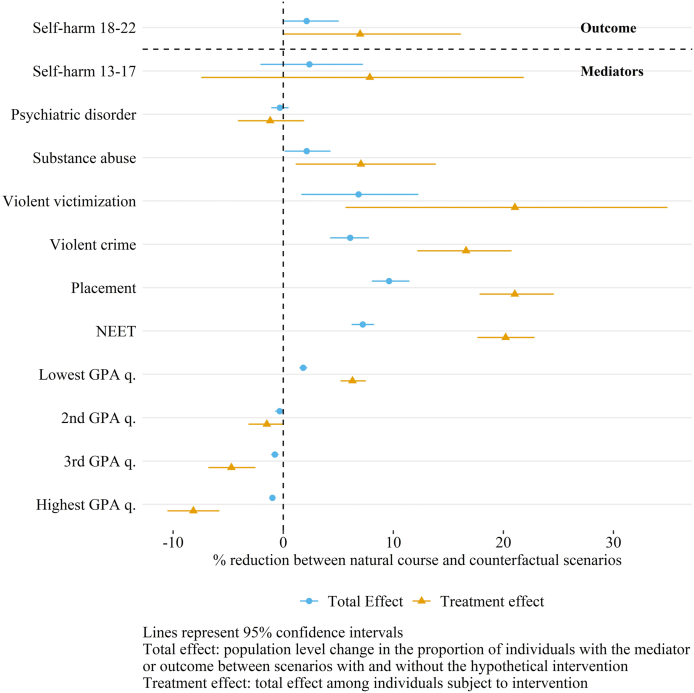
Total effect and average treatment effect for the treated (ATT) of the hypothetical intervention on the outcome and mediators.

At population-level, we found a small 2% decrease between the natural course (NC) and counterfactual (CF) scenarios in the proportion of individuals who self-harmed at least once in young adulthood (Fig. 2). The upper limit of the effect was zero indicating that the effect might not be statistically significant. The average effect of the intervention among the targeted group, the lowest childhood income quintile, was larger. There was a 7% decrease in the proportion of self-harming individuals between the NC and CF scenarios but the upper limit was again practically 0.

The intervention did not have a statistically significant effect on self-harm at age 13–17 but decreased adolescent substance abuse by 2% in the total population and 7% among the treated. The upper limit of 95% confidence interval of the effect was close to 0. The intervention did not impact psychiatric disorders. For the other mediators, we found a clear decrease in the proportion of individuals with violent victimizations (total effect (TE) 7%, average treatment effect for the treated (ATT) 21%), violent crime (TE 6%, ATT 17%), out-of-home placements (TE 10%, ATT 21%) and NEET (TE 7%, ATT 20%). These effects were all statistically significant. In addition, the intervention had an effect on GPA, decreasing the share in the lowest quartile and increasing the shares in the highest two quartiles.

### 3.3 Mediation

We decomposed the total effect into total direct effect (TDE) and natural indirect effect (NIE). The percentages mediated from these decompositions are presented in Table 2. When all the mediators were fixed on natural course values, NIE accounted for 67% of the total effect (TE), and the TDE was not statistically significant (Supplementary files 7 and 8). We then let the intervention impact one mediator at a time (Table 2). Including the effect of previous self-harm, psychiatric disorder, substance abuse or violent victimization in the TDE did not change the percentage mediated. Violent crime, NEET, GPA and especially out-of-home placements lowered the percentage mediated, indicating that these four factors might be more important pathways through which childhood income impacts on self-harm in young adulthood.

**Table 2:** Percentage mediated across different scenarios and 95% confidence intervals.

Direct effect definition	% mediated	95% CI	
TDE	67	62	73
TDE + previous self-harm	65	60	70
TDE + psychiatric disorders	70	65	76
TDE + substance abuse	65	60	70
TDE + victimization	66	61	71
TDE + violent crime	58	54	63
TDE + out-of-home placements	39	36	43
TDE + NEET	53	49	58
TDE + GPA	50	45	54

### 3.4 Subgroup analyses

**Fig. 3:**
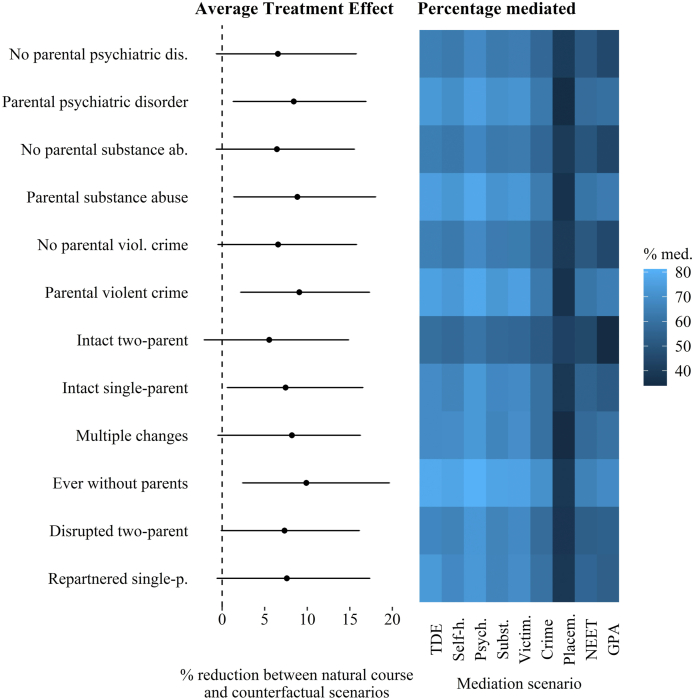
Average treatment effect for the treated (ATT) and percentage mediated in subgroups. Left: ATT. Right: heat map illustrating the percentage mediated across nine different scenarios.

Lastly, we looked into the effect of income on self-harm in young adulthood by childhood family characteristics (parental psychiatric disorders, substance abuse, violent criminality, family stability). The effect of the intervention among those in the lowest childhood income quintile in these subgroups is illustrated in Fig. 3, coupled with a heat map presenting percentage mediated across different definitions of direct effect (Table 2). The distributions of the mediators in these subgroups are available in Supplementary File 8.

The main finding of these subgroup analyses is that among children that have experienced parental substance abuse, psychiatric disorder or violent criminality, the effect is slightly larger than among those without these experiences. Fig. 3 shows a statistically significant decrease of around 8–9% in the proportion of individuals who self-harm in young adulthood across the groups that have experienced these adversities, whereas among those without these experiences the effect is not statistically significant. In the categories of family stability, the effect was highest among those who had lived without parents at any point between age 8 and 12. Moreover, the heat map indicates that among those with adverse childhood experiences, a larger part of the total effect is mediated through the adolescent mediators included in the study.

## 4 Discussion

### 4.1 Main results

We examined the effect of low childhood income on self-harm in young adulthood and the extent to which this effect is mediated through adolescent mental health, behavioural problems and school performance. By assigning a hypothetical intervention of raising all those in the lowest childhood family income quintile to the second-lowest, and adjusting for parental education and several adverse childhood experiences, we found a 2% reduction in the proportion of individuals who self-harmed at least once on the population level and a 7% reduction among those targeted with the intervention, i.e. the individuals in the lowest childhood income quintile. We included a broad range of indicators measuring adolescent mental health, behavioural problems and school performance as mediators, and showed that most (67%) of the total effect of childhood income was mediated by these factors. Our findings refine, more descriptive evidence on the topic (Hawton et al., 2012b, Hawton et al., 2012a; Mok et al., 2018; Page et al., 2014). To our knowledge, this is the first study to quantify the potential population level effects of intervening on low childhood income on self-harm and decomposes the effects into direct and indirect effects.

We interpret childhood income as a “fundamental cause of disease” (Link & Phelan, 1995). Through developmental processes as well as lack of resources (Hodgkinson et al., 2017), low childhood income increases the risk of several, more proximal adolescent risk factors of self-harm (Hawton, Saunders, & O’Connor, 2012). Of these proximal risk factors we included adolescent psychiatric disorders, substance abuse, self-harm, violent victimization, violent crime, out-of-home placements, not being in education, employment and training (NEET) and school performance, and framed these as potential mediating variables. Our main finding shows that most of the effect of income is mediated through these proximal risk factors. We also showed that our hypothetical intervention on income also decreased out-of-home placements, violent victimization, violent crime and NEET, and improved GPA, but had no effect on psychiatric disorders. We are not aware of any previous work, which would have produced similar population-averaged effect estimates on all of these adolescent factors in the same study and thus enabled assessment of the relative contribution of individual mediators.

Our results suggest that previous self-harm, psychiatric disorders, substance abuse or violent victimization have a minor role in mediating the effects of income. However, these factors were shown to be strong independent predictors of self-harm (Table 1), suggesting an increase in the risk of self-harm regardless of childhood income. Out-of-home placements, NEET, GPA and violent crime had stronger contributions to mediation, and were also more clearly associated with childhood income (Supplementary files 5 and 9). Thus, low childhood income seems to operate through the mediators that have a steeper socioeconomic gradient. It is possible that these factors might be a proxy for some other harmful underlying events in adolescence. For instance, alcohol and drug use are associated with out-of-home placements (Hawton et al., 2012b, Hawton et al., 2012a) as well as with NEET (Henderson et al., 2017), and thus the variables may partly reflect these behaviours.

Lastly, we showed that the effect of the hypothetical income intervention was stronger in the subgroups of children that have faced adverse childhood experiences. This indicates that a combination of adverse experiences and living in a low income household are especially harmful (Lanier et al., 2018). In addition, there were indications of larger mediation among children exposed to adverse childhood experiences, which is likely explained by the distribution of mediators: they are more prevalent among the children with adverse childhood experiences than among others (Supplementary file 9). In general, potential mediators have to be sufficiently prevalent in order to be substantially reduced by the intervention and hence for sizeable mediation to occur at the group level.

### 4.2 Policy implications

Our results suggest that increases in childhood income could decrease the risk of self-harm among children from low income families. Besides the direct effect of income, we also showed that most of the effect of the intervention was mediated through other factors in adolescence. The findings are important for designing targeted interventions. Our results suggest that the harmful effects of low family income on the risk of adolescent self-harm might be reduced by targeting adolescents placed into out-of-home care and adolescents with school problems or early violent offences. Paying extensive attention to the mental health needs of the children in these institutional settings – child welfare services, schools and the police – could effectively prevent self-harm in young adulthood.

### 4.3 Strengths and limitations

There are several strengths in this study. Our data is an annually updated population-level dataset, with little attrition or non-response, a long follow-up and no recall bias. The data allow for the identification of adolescents and young adults with mental health conditions, substance abuse, self-harm, crime and victimization as well as their school performance, and similar information for their parents. These individuals and families may be hard to reach with population-level surveys, giving us a unique opportunity to study adolescent disadvantage. Moreover, as the information is obtained from several different sources, the data available are diverse and complementary. Moreover, our choice of method using counterfactual scenarios offers new insights into the population-level effects of childhood income on self-harm and the magnitude of mediation of these effects through other adolescent factors.

As with any study, there are also limitations. First, administrative data underestimates the true prevalence of the health and behavioural problems investigated and limits the examination to more severe cases. However, we had access to specialized outpatient health care data, which includes, to some extent, less severe episodes. Nevertheless, we might underestimate the prevalence and effects of these disorders among children from poor families, if there are differences in treatment-seeking behaviour and access to health care between better- and worse-off families. However, in Finland, income is not a major barrier for treatment-seeking (Junna et al., 2019). The quality of the Finnish health care data have been evaluated elsewhere (Sund, 2012), and is shown to have excellent coverage, except for psychiatric outpatient visits during the earliest years of observation. However, since we use five-year age bands, inpatient and outpatient data and the earliest years of observation are only used for parents, we feel confident that incomplete data should not bias our results. Furthermore, given that contextual factors and system-level characteristics have stayed similar, strong cohort effects appear unlikely.

Second, the parametric g-formula has its limitations in the case of rare events. When the outcome is rare, the population-averaged effect sizes are very small, especially after decomposing effects (Supplementary file 7 and 8). The small effect sizes and total direct effect fluctuating around zero meant that the calculation of percentage mediated via Monte Carlo integration was unstable and the results uninformative. Instead, we calculated percentage mediated as the ratio of average indirect effect and average total effect. This may introduce bias, as the average of a set of ratios does not necessarily equal the ratio of a set of averages. However, reassuringly, the median of percentage mediated in the bootstrap iterations was 65%, which is very close to our estimation (67%). Moreover, our simulation study and closer evaluation of the effect distributions (Supplementary file 9) further confirm that the g-formula is applicable for rare events.

As this study presents causal claims, the assumptions of positivity, exchangeability and consistency underlying causal identification need to be discussed. We provide a more in-depth discussion on these assumption in Supplementary File 2. On the one hand, we note that it is likely that some unmeasured confounding may still affect our results, which would bias our effect estimates upwards. Unmeasured parental alcohol abuse, to the extent that it does not lead to measured parental psychiatric disorders, substance abuse and violent crime, might bias the effect of income on self-harm identified upward. On the other hand, we hold the confounders constant at their initial observed values, which could dampen the effect of income if the situations in families improve due to intervention. Implementing our hypothesized intervention might also be unrealistic and have unintended consequences if applied in reality. For instance, increasing income of one group might lead to downward social mobility in others, which has the potential to increase mental health problems and self-harm (Hawton et al., 2012b, Hawton et al., 2012a).Therefore, causal interpretation of our results requires caution.

## 5 Conclusions

In this study, we showed that living in a low income household during childhood increases the risk of self-harm in young adulthood, as well as the risk of adolescent mental health, behavioural problems and school performance. Our results suggest that most of the effect of low childhood income on self-harm is mediated through events and experiences occurring in adolescence, in particular out-of-home placements. These findings both refine the existing evidence and shed light on previously less known aspects in the association between childhood income and self-harm. We encourage future applications of the parametric g-formula in self-harm research. Examinations of time-varying confounding, mediation and outcomes as well as other types of hypothetical interventions might further elucidate the complex interplay between self-harming behavior, family characteristics and individual factors. In settings without time-varying exposures, other mediation methods can also be used. We have described a number of potential advantages of the g-formula over other methods, but an interesting avenue of future research could be a close comparison of methods in this setting.

## Ethical statement

The study has been approved by Statistics Finland Board of Statistical Ethics (TK-53-1121-18). Permissions to use the data were granted by each register holder. The data used were anonymized and participants were not contacted. In Finland, informed consent is not required for register-based studies.

## CRediT authorship contribution statement

**Joonas Pitkänen:** Formal analysis, Writing – original draft, Conceptualization. **Maarten J. Bijlsma:** Methodology, Writing – original draft, Conceptualization. **Hanna Remes:** Writing – original draft, Conceptualization. **Mikko Aaltonen:** Writing – original draft, Conceptualization.

## Declaration of competing interest

None.
