# Supplementary material for: The effect of low childhood income on self-harm in young adulthood: Mediation by adolescent mental health, behavioural factors and school performance
Source: SSM Popul Health. 2021 Feb 18;13:100756. doi: 10.1016/j.ssmph.2021.100756 (PMC7910518; doi:10.1016/j.ssmph.2021.100756)
Supplement: Multimedia component 1 [file mmc1.pdf]

The effect of low childhood income on self-harm in young adulthood: mediation by adolescent mental health, behavioural factors and school performance.

Supplementary Material

1/14/2021

Contents

Supplementary File 1: ICD10-codes used to define variables **2**

Supplementary File 2: Model theoretical validity and assumptions for causal identification **3**

Supplementary File 3: G-formula step-by-step **5**

Supplementary File 4: Cleaned and cut R code **6**

Supplementary File 5: The underlying models for the outcome and mediators used in g-formula simulations. **18**

Supplementary file 6: Natural course means and observed means of the outcome and mediators **22**

Supplementary file 7: Absolute effect sizes among the total population and the treated **23**

Supplementary file 8: Evaluating rare outcomes **25**

Supplementary file 9: Distributions of mediators (as percentages) in the subgroups used in subgroup analyses **38**

Supplementary File 1: ICD10-codes used to define variables

Psychiatric disorders

F20-69, F80-99

Substance abuse

F10-16, F18-19, R780-85, T40, T423-4, T426-7, T430-6, T438-9, T507, T51, Z502-3, Z714-5, Z721-2, X45, Y90-1 (the last three are identified from external causes of injuries and poisonings).

Violent victimization

X85-99, Y00-9

Supplementary File 2: Model theoretical validity and assumptions for causal identification

Model

In this study, we assumed that childhood income has an effect on self-harm in young adulthood both directly and indirectly through several mediating variables measured during adolescence. We further assumed that factors in childhood may confound these associations. Factors examined in this study are likely to form rather complex time-varying interplay, characterized by interdependencies, accumulation, multidirectional associations and interactions. However, due to discrepancies in the accuracy (date-level vs. annual measurements) and the nature of the data, the correct timing and sequences of different events are hard if not impossible to determine. Moreover, many of the factors studied in the paper are considerably rare, making the data quickly sparse in complex panel settings. Therefore, we were forced to simplify our model to identify the impact of income on self-harm and the pathways through which it might be mediated.

We simplified the pathways by dividing the life-course between age 8 and 22 into five-year age bands as follows: age 8–12 for childhood, 13–17 for adolescence and 18–22 for young adulthood. In the measurement of the variables, we only take into account those events occurring during these age bands. Hence, childhood income and confounders are measured at age 8–12, mediators at age 13–17 and outcome at age 18–22, lessening the time-related overlap between these interdependent processes. All the variables are treated as independent predictors in the models used. We acknowledge that treating the variables as time-invariant and mutually adjusting for all the mediators and confounders may introduce bias to our estimation. However, despite the theoretical flaws of the model, the simplification enabled us to identify both direct and indirect effects and to include a more diverse range of mediators, thus meeting the aim of our study better than a cross-lagged model. We encourage future researchers to implement more sophisticated models, where possible.

Assumptions for causal identification

Use of g-formula relies on the three fundamental assumptions of identifying causal effects. First, the positivity assumption requires that each individual would have the possibility of receiving the treatment assigned (Platt et al., 2012). In the current study, this would mean that all the children in the lowest income quintile could theoretically be moved to the second income quintile. In our view, there is no violation of this assumption.

Second, the exchangeability assumption requires that children in the lowest income quintile, conditional on the measured covariates, are comparable to children in the other quintiles in regard to their risk of the outcome (Greenland and Robins, 2009). We controlled for confounding of several childhood factors but it seems likely that there could be other factors present that could act as meaningful confounders but which we cannot identify within our data. For instance, unmeasured childhood adversity may strengthen the effect of childhood income on self-harm, as these experiences are more prevalent in low-income groups (Halfon et al. 2017). On the other hand, we hold parental alcohol and substance abuse, psychiatric disorders and violent crime constant in the presence of increasing income, which may lead to overcontrolling, as increases in income might improve these conditions (Kröger et al., 2015).

Third, the consistency assumption requires that the exposure variable of interest and the designed intervention are precisely defined (Rehkopf et al., 2016). Our exposure of interest in this study was childhood income, which was defined as a five-year mean and divided into quintiles. A quintile-based approach allowed us to intuitively categorize individuals, while still maintaining a sizable number of individuals experiencing the outcome in each group. Intervening on this variable is straightforward and the effects can be attributed to income. However, the intervention may not be realistic, and would require e.g. strong governmental actions. Moreover, raising children from the lowest to the second-lowest quintile also implies that the actual income distribution would change. Given that downward social mobility may increase self-harm (Mok et al., 2018), there might be bias due to violation of the consistency assumption. Therefore, causal interpretation of our results warrants caution.

Greenland, S., Robins, J.M., 2009. Identifiability, exchangeability and confounding revisited. Epidemiol Perspect Innov 6, 4. https://doi.org/10.1186/1742-5573-6-4

Halfon, N., Larson, K., Son, J., Lu, M., Bethell, C., 2017. Income Inequality and the Differential Effect of Adverse Childhood Experiences in US Children. Academic Pediatrics, Child Well-Being and Adverse Childhood Experiences in the US 17, S70–S78. https://doi.org/10.1016/j.acap.2016.11.007

Kröger, H., Pakpahan, E., Hoffmann, R., 2015. What causes health inequality? A systematic review on the relative importance of social causation and health selection. Eur J Public Health 25, 951–960. https://doi.org/10.1093/eurpub/ckv111

Mok, P.L.H., Antonsen, S., Pedersen, C.B., Carr, M.J., Kapur, N., Nazroo, J., Webb, R.T., 2018. Family income inequalities and trajectories through childhood and self-harm and violence in young adults: a population-based, nested case-control study. The Lancet Public Health 3, e498–e507. https://doi.org/10.1016/S2468-2667(18)30164-6

Platt, R.W., Delaney, J.A.C., Suissa, S., 2012. The positivity assumption and marginal structural models: the example of warfarin use and risk of bleeding. European Journal of Epidemiology 27, 77–83. Rehkopf, D.H., Glymour, M.M., Osypuk, T.L., 2016. The Consistency Assumption for Causal Inference in Social Epidemiology: When a Rose is Not a Rose. Curr Epidemiol Rep 3, 63–71. https://doi.org/10.1007/s40471-016-0069-5

Supplementary File 3: G-formula step-by-step

1. Sample with replacement the original amount of individuals from the data.
2. Use this resampled data to fit suitable models for each mediator and outcome.
3. Using the resampled data and the fitted models, obtain predicted probabilities and with these probabilities, draw new values from binomial distribution (or some other distribution used to model the variables) and create a new data set with these new values (We refer to this process as simulation below). Simulate mediators first and then use these simulated values in the model for the outcome. This is the natural course (NC) step.
4. Copy the resampled data set (from step 1) and introduce your planned intervention by changing the relevant covariate values (e.g. income quintile).
5. Using this data and the fitted models, simulate a new data set containing new values produced in the presence of the intervention, similar to step 3. This is the counterfactual (CF) step.
6. Copy the resampled data (from step 1), and draw mediator values from the NC and intervene in the data as in step 5. Simulate new values for the outcome using this data. This definition corresponds to the Total Direct Effect (TDE) and Natural Indirect Effect (NIE) definition. Other mediation scenarios are possible (see e.g. Wang & Arah 2015).
7. Optional: to examine pathways, produce a new set of data and let the intervention impact a set of mediators at a time.
8. Save the averages of the variables of interest in each of the newly produced sets of data. These averages are the proportions of individuals with given outcome/mediator, if the variables are binary or categorical.
9. Repeat steps 3–7 X number of times to reduce Monte Carlo Error, where X is a value large enough to produce stable estimates.
10. Average over the means produced in step 9 to achieve a stable estimator for expected value of each of the variables in each of the scenarios.
11. Calculate effects. Subtract the stabilized NC mean from the stabilized CF mean to achieve Total Effect. Decompose the effect as instructed in the literature. For instance, TDE is achieved by subtracting the natural course mean from the mediation course mean, and NIE is then achieved by subtracting TDE from TE.
12. For relative effects, calculate ratios between the scenarios. For instance, relative TE is the ratio of CF and NC estimates. Proportion mediated is calculated as the ratio of IE to TE.
13. Save all the effects. If needed, save effects by subgroup.
14. Repeat the above steps K times to produce bootstrap confidence intervals for the effects, and save all the effects produced in each bootstrap, where K is a large enough value (100+) to produce stable estimates.
15. Calculate the final effects by averaging over the bootstrap iterations, and confidence intervals as the 2.5th and 97.5th quantile of the distribution.

Wang, A., Arah, O.A., 2015. G-computation demonstration in causal mediation analysis. Eur J Epidemiol 30, 1119–1127. https://doi.org/10.1007/s10654-015-0100-z

Supplementary File 4: Cleaned and cut R code

```
#The effect of low childhood income on self-harm in young adulthood: mediation
#by adolescent mental health, behavioural factors and school performance.

#The g-foirmula algorithm
#Modified from Bijlsma et al. 2019

#Cleaned and annotated version
#Free to use with acknowledgments to authors

setwd(...)
selfharm.dat <- read(...) #Read in the data

#Preparations before the start of the actual algorithm

#Expit-function to be used for transforming predictions
expit <- function(x) {exp(x)/(1+exp(x))}

#Function for categorical predictions
multinom.4var.predict <- function(
  object.cat1,object.cat2,object.cat3,object.cat4,newdata) {

  # make predictions and turn them into probabilities
  p1 <- expit(predict(object.cat1,newdata=newdata))
  p2 <- expit(predict(object.cat2,newdata=newdata))
  p3 <- expit(predict(object.cat3,newdata=newdata))
  p4 <- expit(predict(object.cat4,newdata=newdata))

  # join the probabilities in a matrix
  # where nrow is the number of categories
  x <- t(matrix(rbind(p1,p2,p3,p4),nrow=4))

  # draw predictions for each row from a multinomial distributon
  # using the probabilities just estimated
  x <- t(apply(x,1,rmultinom,n=1,size=1))

  # return the output
  return(x)
}


#Data transformations if needed


#Formulas for mediators

formula.mediator <- c(
  "outcome ~ inc1 + inc2 + inc3 + inc4 +
paredu1 + paredu2 + fam2 + fam3 + fam4 + fam5 + fam6 +
female + b_1990 + b_1991 + b_1992 + b_1993 + b_1994 +
parcrime + parsub + parpsy")
```

```r
formula.SUB <- as.formula(sub('outcome','sub_1317',formula.mediator))
formula.PSY <- as.formula(sub('outcome','psy_1317',formula.mediator))
formula.SFH <- as.formula(sub('outcome','sfh_1317',formula.mediator))
formula.VIOL <- as.formula(sub('outcome','viol_1317',formula.mediator))
formula.violent_crime <- as.formula(sub('outcome','cri_1317',formula.mediator))
formula.neet <- as.formula(sub('outcome','NEET',formula.mediator))
formula.placement <- as.formula(sub('outcome','placement',formula.mediator))
formula.gpa1 <- as.formula(sub('outcome','lowest_gpa',formula.mediator))
formula.gpa2 <- as.formula(sub('outcome','scnd_gpa2',formula.mediator))
formula.gpa3 <- as.formula(sub('outcome','trd_gpa',formula.mediator))
formula.gpa4 <- as.formula(sub('outcome','frth_gpa',formula.mediator))

#########################################
# Arrays for saving MC results
#########################################
msize <- 100 #Monte carlo iterations
bssize <- 250 #bootstrap iterations
med.scenarios <- 2 #TDE and TDE + plcements

#Number of variables of interest: 12
nvar <- 12

#Number of groups:
#Full population +
#Subgroups: 1. treated, 2-3. genders separately,

ngroups <- 4

#Array for natural course and counterfactual
#means of all the variables and by subgroup

nc.array <- rep(NA,msize*nvar*ngroups)
dim(nc.array) <- c(msize,nvar,ngroups)

cf.array <- nc.array

#Make an array, where to save the
#scenario means among th treated in subgroups

#group size=2, as the total population and treated (low income=1)
#don't need to be included

att.nc.array <- rep(NA,msize*nvar*2)
dim(att.nc.array) <- c(msize,nvar,2)

att.cf.array <- att.nc.array

#Arrays for mediation scenario means of self-harm
#Save these for the subgroups too

med.array <- rep(NA,msize*med.scenarios*ngroups)
dim(med.array) <- c(msize,med.scenarios,ngroups)
```

```
#######################################
# Arrays for saving bootstrap results
#######################################
#Natural course and counterfactual

bs.nc.array <- rep(NA,bssize*nvar*ngroups)
dim(bs.nc.array) <- c(bssize,nvar,ngroups)
bs.cf.array <- bs.nc.array

#Arrays for those that are treated in the subgroups

bs.att.nc.array <- rep(NA,bssize*nvar*2)
dim(bs.att.nc.array) <- c(bssize,nvar,2)
bs.att.cf.array <- bs.att.nc.array

#Mediation scenario arrays
bs.tde.array <- rep(NA,bssize*med.scenarios*ngroups)
dim(bs.tde.array) <- c(bssize,med.scenarios,ngroups)

#################################
#Arrays for effect calculations
#################################

#Te for all the variables + subgroup
te.bs.array <- rep(NA,bssize*nvar*ngroups)
dim(te.bs.array) <- c(bssize,nvar,ngroups)

#Treatment effect in subgroups
att.bs.array <- rep(NA,bssize*nvar*2)
dim(att.bs.array) <- c(bssize,nvar,2)

#Make copies for relative effect
te.rel.bs.array <- te.bs.array
att.rel.bs.array <- att.bs.array

#Copies of mediation array
#and for IE and ratios
bs.ie.array <- bs.tde.array
bs.perc.med.array <- bs.tde.array


###############
#The g-formula#
###############

for(bs in 1:bssize) {

  n <- dim(selfharm.dat)[1] # save sample size
  index <- sample(n,replace=T) # sample with replacement
  sample.dat <- selfharm.dat[index,]

  #Fit all the mediator models using the sampled data
```

```
fit.sub <- glm(formula.SUB,family='binomial',data=sample.dat)
fit.sfh <- glm(formula.SFH,family='binomial',data=sample.dat)
fit.psy <- glm(formula.PSY,family='binomial',data=sample.dat)
fit.crime <- glm(formula.violent_crime,family='binomial',data=sample.dat)
fit.vict<- glm(formula.VIOL,family='binomial',data=sample.dat)
fit.NEET <- glm(formula.neet,family='binomial',data=sample.dat)
fit.placement <- glm(formula.placement,family='binomial',data=sample.dat)
fit.gpa1 <- glm(formula.gpa1,family='binomial',data=sample.dat)
fit.gpa2 <- glm(formula.gpa2,family='binomial',data=sample.dat)
fit.gpa3 <- glm(formula.gpa3,family='binomial',data=sample.dat)
fit.gpa4 <- glm(formula.gpa4,family='binomial',data=sample.dat)

#Fit model for outcome

fit.selfharm <- glm(sfh_1822 ~ inc1 + inc2 + inc3 + inc4 +
                      paredu1 + paredu2 + female +
                      b_1990 + b_1991 + b_1992 + b_1993 + b_1994 +
                      parcrime + parsub + parpsy +
                      fam2 + fam3 + fam4 + fam5 + fam6 +
                      sfh_1317 + psy_1317 + sub_1317 + viol_1317 + cri_1317 +
                      NEET + placement +
                      lowest_gpa + scnd_gpa2 + trd_gpa,
                    family=binomial, data=sample.dat)
#Subgroup ids
treated.id <- unique(sample.dat$id[sample.dat$tulo1==1])
female.id <- unique(sample.dat$id[sample.dat$female==1])
male.id <- unique(sample.dat$id[sample.dat$female==0])

#Treated in subgroups

tr.female.id <- unique(sample.dat$id[sample.dat$female==1 & sample.dat$tulo1==1])
tr.male.id <- unique(sample.dat$id[sample.dat$female==0 & sample.dat$tulo1==1])

subgroups <- list(treated.id,female.id,male.id)

treated.subgroups <- list(tr.female.id,tr.male.id)

#Start predictions
#This code uses loops to reduce MC error

for(m in 1:msize) {
  #####################
  #Natural course step#
  #####################

  #Copy data
  sample.xMx <- sample.dat

  #Replace mediator values with newly predicted values

  sample.xMx$psy_1317 <- rbinom(n,1,expit(
    predict(object=fit.psy,newdata=sample.xMx)))
  sample.xMx$sfh_1317 <- rbinom(n,1,expit(
```

```r
  predict(object=fit.sfh,newdata=sample.xMx)))
sample.xMx$sub_1317 <- rbinom(n,1,expit(
  predict(object=fit.sub,newdata=sample.xMx)))
sample.xMx$viol_1317 <- rbinom(n,1,expit(
  predict(object=fit.vict,newdata=sample.xMx)))
sample.xMx$cri_1317 <- rbinom(n,1,expit(
  predict(object=fit.crime,newdata=sample.xMx)))
sample.xMx$NEET <- rbinom(n,1,expit(
  predict(object=fit.NEET,newdata=sample.xMx)))
sample.xMx$placement <- rbinom(n,1,expit(
  predict(object=fit.placement,newdata=sample.xMx)))

x <-  multinom.4var.predict(fit.gpa1,fit.gpa2,fit.gpa3,fit.gpa4,sample.xMx)
sample.xMx$lowest_gpa <- x[,1]
sample.xMx$scnd_gpa2 <- x[,2]
sample.xMx$trd_gpa <- x[,3]
sample.xMx$frth_gpa <- x[,4]

#Lastly, predict new vaues for the outcome

sample.xMx$sfh_1822 <- rbinom(n,1,expit(
  predict(object=fit.selfharm,newdata=sample.xMx)))

#####################
#Counterfactual step#
#####################

#Copy the sampled data

sample.xstarMxstar <- sample.dat

#Make an intervention
sample.xstarMxstar$inc2 <-
  ifelse(sample.xstarMxstar$inc1==1,1,sample.xstarMxstar$inc2)
sample.xstarMxstar$inc1 <- 0

#Predict new values for mediators
sample.xstarMxstar$psy_1317 <- rbinom(n,1,expit(
  predict(object=fit.psy,newdata=sample.xstarMxstar)))
sample.xstarMxstar$sfh_1317 <- rbinom(n,1,expit(
  predict(object=fit.sfh,newdata=sample.xstarMxstar)))
sample.xstarMxstar$sub_1317 <- rbinom(n,1,expit(
  predict(object=fit.sub,newdata=sample.xstarMxstar)))
sample.xstarMxstar$viol_1317 <- rbinom(n,1,expit(
  predict(object=fit.vict,newdata=sample.xstarMxstar)))
sample.xstarMxstar$cri_1317 <- rbinom(n,1,expit(
  predict(object=fit.crime,newdata=sample.xstarMxstar)))
sample.xstarMxstar$NEET <- rbinom(n,1,expit(
  predict(object=fit.NEET,newdata=sample.xstarMxstar)))
sample.xstarMxstar$placement <- rbinom(n,1,expit(
  predict(object=fit.placement,newdata=sample.xstarMxstar)))

x <-  multinom.4var.predict(fit.gpa1,fit.gpa2,fit.gpa3,fit.gpa4,sample.xstarMxstar)
```

```
sample.xstarMxstar$lowest_gpa <- x[,1]
sample.xstarMxstar$scnd_gpa2 <- x[,2]
sample.xstarMxstar$trd_gpa <- x[,3]
sample.xstarMxstar$frth_gpa <- x[,4]

#Predict new values for the outcome

sample.xstarMxstar$sfh_1822 <- rbinom(n,1,expit(
  predict(object=fit.selfharm,newdata=sample.xstarMxstar)))

#####################
#Total direct effect#
#####################

#Copy data
sample.xstarMx <- sample.dat

#Make the same intervention
sample.xstarMx$inc2 <-
  ifelse(sample.xstarMx$inc1==1,1,sample.xstarMx$inc2)
sample.xstarMx$inc1 <- 0

#Draw mediator values from the Natural Course
sample.xstarMx$psy_1317 <- sample.xMx$psy_1317
sample.xstarMx$sfh_1317 <- sample.xMx$sfh_1317
sample.xstarMx$sub_1317 <- sample.xMx$sub_1317
sample.xstarMx$viol_1317 <- sample.xMx$viol_1317
sample.xstarMx$cri_1317 <- sample.xMx$cri_1317
sample.xstarMx$NEET <- sample.xMx$NEET
sample.xstarMx$placement <- sample.xMx$placement
sample.xstarMx$lowest_gpa <- sample.xMx$lowest_gpa
sample.xstarMx$scnd_gpa2 <- sample.xMx$scnd_gpa2
sample.xstarMx$trd_gpa <- sample.xMx$trd_gpa
sample.xstarMx$frth_gpa <- sample.xMx$frth_gpa

#Predict outcome
sample.xstarMx$sfh_1822 <- rbinom(n,1,expit(
  predict(object=fit.selfharm,newdata=sample.xstarMx)))

##################
#TDE + placements#
##################

#Copy data
sample.xstarMxfam <- sample.dat

#Intervene
sample.xstarMxfam$inc2 <-
  ifelse(sample.xstarMxfam$inc1==1,1,sample.xstarMxfam$inc2)
sample.xstarMxfam$inc <- 0

#Draw otther mediators from the NC
```

```r
sample.xstarMxfam$sfh_1317 <- sample.xMx$sfh_1317
sample.xstarMxfam$sub_1317 <- sample.xMx$sub_1317
sample.xstarMxfam$viol_1317 <- sample.xMx$viol_1317
sample.xstarMxfam$cri_1317 <- sample.xMx$cri_1317
sample.xstarMxfam$NEET <- sample.xMx$NEET
sample.xstarMxfam$psy_1317 <- sample.xMx$psy_1317
sample.xstarMxfam$lowest_gpa <- sample.xMx$lowest_gpa
sample.xstarMxfam$scnd_gpa2 <- sample.xMx$scnd_gpa2
sample.xstarMxfam$trd_gpa <- sample.xMx$trd_gpa
sample.xstarMxfam$frth_gpa <- sample.xMx$frth_gpa

#Predict the mediator of interest
sample.xstarMxfam$placement <- sample.xstarMxstar$placement

#Predict the outcome

sample.xstarMxfam$sfh_1822 <- rbinom(n,1,expit(
  predict(object=fit.selfharm,newdata=sample.xstarMxfam)))

## save means of the current mc loop
#in all the subgroups

nc.array[m,1,1] <- mean(sample.xMx$sfh_1822)
nc.array[m,2,1] <- mean(sample.xMx$sfh_1317)
nc.array[m,3,1] <- mean(sample.xMx$psy_1317)
nc.array[m,4,1] <- mean(sample.xMx$sub_1317)
nc.array[m,5,1] <- mean(sample.xMx$viol_1317)
nc.array[m,6,1] <- mean(sample.xMx$cri_1317)
nc.array[m,7,1] <- mean(sample.xMx$NEET)
nc.array[m,8,1] <- mean(sample.xMx$placement)
nc.array[m,9,1] <- mean(sample.xMx$lowest_gpa)
nc.array[m,10,1] <- mean(sample.xMx$scnd_gpa2)
nc.array[m,11,1] <- mean(sample.xMx$trd_gpa)
nc.array[m,12,1] <- mean(sample.xMx$frth_gpa)

cf.array[m,1,1] <- mean(sample.xstarMxstar$sfh_1822)
cf.array[m,2,1] <- mean(sample.xstarMxstar$sfh_1317)
cf.array[m,3,1] <- mean(sample.xstarMxstar$psy_1317)
cf.array[m,4,1] <- mean(sample.xstarMxstar$sub_1317)
cf.array[m,5,1] <- mean(sample.xstarMxstar$viol_1317)
cf.array[m,6,1] <- mean(sample.xstarMxstar$cri_1317)
cf.array[m,7,1] <- mean(sample.xstarMxstar$NEET)
cf.array[m,8,1] <- mean(sample.xstarMxstar$placement)
cf.array[m,9,1] <- mean(sample.xstarMxstar$lowest_gpa)
cf.array[m,10,1] <- mean(sample.xstarMxstar$scnd_gpa2)
cf.array[m,11,1] <- mean(sample.xstarMxstar$trd_gpa)
cf.array[m,12,1] <- mean(sample.xstarMxstar$frth_gpa)

#Subgroups

for(sg in 1:3){
  current.sg <- unlist(subgroups[sg]) # I take all the subgroups one by one
  current.nc <- sample.xMx[which(sample.xMx$id %in% current.sg),]
```

```
  current.cf <- sample.xstarMxstar[which(sample.xstarMxstar$id %in% current.sg),]

  nc.array[m,1,sg+1] <- mean(current.nc$sfh_1822) #+1 since the 1st matrix includes all ind.
  nc.array[m,2,sg+1] <- mean(current.nc$sfh_1317)
  nc.array[m,3,sg+1] <- mean(current.nc$psy_1317)
  nc.array[m,4,sg+1] <- mean(current.nc$sub_1317)
  nc.array[m,5,sg+1] <- mean(current.nc$viol_1317)
  nc.array[m,6,sg+1] <- mean(current.nc$cri_1317)
  nc.array[m,7,sg+1] <- mean(current.nc$NEET)
  nc.array[m,8,sg+1] <- mean(current.nc$placement)
  nc.array[m,9,sg+1] <- mean(current.nc$lowest_gpa)
  nc.array[m,10,sg+1] <- mean(current.nc$scnd_gpa2)
  nc.array[m,11,sg+1] <- mean(current.nc$trd_gpa)
  nc.array[m,12,sg+1] <- mean(current.nc$frth_gpa)

  cf.array[m,1,sg+1] <- mean(current.cf$sfh_1822)
  cf.array[m,2,sg+1] <- mean(current.cf$sfh_1317)
  cf.array[m,3,sg+1] <- mean(current.cf$psy_1317)
  cf.array[m,4,sg+1] <- mean(current.cf$sub_1317)
  cf.array[m,5,sg+1] <- mean(current.cf$viol_1317)
  cf.array[m,6,sg+1] <- mean(current.cf$cri_1317)
  cf.array[m,7,sg+1] <- mean(current.cf$NEET)
  cf.array[m,8,sg+1] <- mean(current.cf$placement)
  cf.array[m,9,sg+1] <- mean(current.cf$lowest_gpa)
  cf.array[m,10,sg+1] <- mean(current.cf$scnd_gpa2)
  cf.array[m,11,sg+1] <- mean(current.cf$trd_gpa)
  cf.array[m,12,sg+1] <- mean(current.cf$frth_gpa)

}

#means for treated among subgroups

for(sg in 1:2){
  current.sg <- unlist(treated.subgroups[sg])
  current.nc <- sample.xMx[which(sample.xMx$id %in% current.sg),]
  current.cf <- sample.xstarMxstar[which(sample.xstarMxstar$id %in% current.sg),]

  att.nc.array[m,1,sg] <- mean(current.nc$sfh_1822)
  att.nc.array[m,2,sg] <- mean(current.nc$sfh_1317)
  att.nc.array[m,3,sg] <- mean(current.nc$psy_1317)
  att.nc.array[m,4,sg] <- mean(current.nc$sub_1317)
  att.nc.array[m,5,sg] <- mean(current.nc$viol_1317)
  att.nc.array[m,6,sg] <- mean(current.nc$cri_1317)
  att.nc.array[m,7,sg] <- mean(current.nc$NEET)
  att.nc.array[m,8,sg] <- mean(current.nc$placement)
  att.nc.array[m,9,sg] <- mean(current.nc$lowest_gpa)
  att.nc.array[m,10,sg] <- mean(current.nc$scnd_gpa2)
  att.nc.array[m,11,sg] <- mean(current.nc$trd_gpa)
  att.nc.array[m,12,sg] <- mean(current.nc$frth_gpa)

  att.cf.array[m,1,sg] <- mean(current.cf$sfh_1822)
  att.cf.array[m,2,sg] <- mean(current.cf$sfh_1317)
  att.cf.array[m,3,sg] <- mean(current.cf$psy_1317)
```

```
    att.cf.array[m,4,sg] <- mean(current.cf$sub_1317)
    att.cf.array[m,5,sg] <- mean(current.cf$viol_1317)
    att.cf.array[m,6,sg] <- mean(current.cf$cri_1317)
    att.cf.array[m,7,sg] <- mean(current.cf$NEET)
    att.cf.array[m,8,sg] <- mean(current.cf$placement)
    att.cf.array[m,9,sg] <- mean(current.cf$lowest_gpa)
    att.cf.array[m,10,sg] <- mean(current.cf$scnd_gpa2)
    att.cf.array[m,11,sg] <- mean(current.cf$trd_gpa)
    att.cf.array[m,12,sg] <- mean(current.cf$frth_gpa)

  }

  #Then, the mediation scenario means
  #Column=scenario
  #Matrix=subgroup
  med.array[m,1,1] <- mean(sample.xstarMx$sfh_1822) #TDE
  med.array[m,2,1] <- mean(sample.xstarMxfam$sfh_1822) #TDE + placement


  for(sg in 1:3){
    current.sg <- unlist(subgroups[sg])
    current.nc <- sample.xMx[which(sample.xMx$id %in% current.sg),]
    current.tde <- sample.xstarMx[which(sample.xstarMx$id %in% current.sg),]
    current.tdefam <- sample.xstarMxfam[which(sample.xstarMxfam$id %in% current.sg),]

    med.array[m,1,sg+1] <- mean(current.tde$sfh_1822)
    med.array[m,2,sg+1] <- mean(current.tdefam$sfh_1822)

  }

  #Repeat this until estimates stabilize (20-100 times)

}

#After all the MC iterations,
#save the bootstrap results


#Calculate effects

##############
#Total effect#
##############

for(v in 1:nvar){

  #Here, the matrix number is again subgroup
  #Column number is the variable of interest
  #Average over the MC iterations to get a stable estimate
  #For the particular bootstrap iteration

  te.bs.array[bs,v,1] <- mean(cf.array[,v,1])-mean(nc.array[,v,1])
  te.bs.array[bs,v,2] <- mean(cf.array[,v,2])-mean(nc.array[,v,2])
```

```
  te.bs.array[bs,v,3] <- mean(cf.array[,v,3])-mean(nc.array[,v,3])
  te.bs.array[bs,v,4] <- mean(cf.array[,v,4])-mean(nc.array[,v,4])

  #Save the relative effect here, too.

  te.rel.bs.array[bs,v,1] <- mean(cf.array[,v,1])/mean(nc.array[,v,1])
  te.rel.bs.array[bs,v,2] <- mean(cf.array[,v,2])/mean(nc.array[,v,2])
  te.rel.bs.array[bs,v,3] <- mean(cf.array[,v,3])/mean(nc.array[,v,3])
  te.rel.bs.array[bs,v,4] <- mean(cf.array[,v,4])/mean(nc.array[,v,4])

  #Then the interactionish part, which again the means among the treated
  #in each subgroup

  att.bs.array[bs,v,1] <- mean(att.cf.array[,v,1])-mean(att.nc.array[,v,1])
  att.bs.array[bs,v,2] <- mean(att.cf.array[,v,2])-mean(att.nc.array[,v,2])


  att.rel.bs.array[bs,v,1] <- mean(att.cf.array[,v,1])/mean(att.nc.array[,v,1])
  att.rel.bs.array[bs,v,2] <- mean(att.cf.array[,v,2])/mean(att.nc.array[,v,2])

}
#That completes the Total effect calculations

#Next, calculate the total direct effect
#This is done by subtracting the NC mean
#from the mean of each mediation scenario

#Here, s=scenario number
#Matrix number is the subgroup
#Column=1 in the nc.array is the column where self-harm is located

for(s in 1:9){
  bs.tde.array[bs,s,1] <- mean(med.array[,s,1]) - mean(nc.array[,1,1])
  bs.tde.array[bs,s,2] <- mean(med.array[,s,2]) - mean(nc.array[,1,2])
  bs.tde.array[bs,s,3] <- mean(med.array[,s,3]) - mean(nc.array[,1,3])
  bs.tde.array[bs,s,4] <- mean(med.array[,s,4]) - mean(nc.array[,1,4])

  #Then, the indirect effect is calculated by subtracting
  #the TDE from the TE: IE=TE-TDE
  #The TE is in the first column of the bootstrap array
  #For each subgroup
  #te.bs.array[bs,1,sg]
  #The TDE is located in the array above
  #bs.tde.array[bs,s,sg], where s is the current scenario
  #Matrix number is the subgroup

  bs.ie.array[bs,s,1] <- te.bs.array[bs,1,1]-bs.tde.array[bs,s,1]
  bs.ie.array[bs,s,2] <- te.bs.array[bs,1,2]-bs.tde.array[bs,s,2]
  bs.ie.array[bs,s,3] <- te.bs.array[bs,1,3]-bs.tde.array[bs,s,3]
  bs.ie.array[bs,s,4] <- te.bs.array[bs,1,4]-bs.tde.array[bs,s,4]

  #Percentage mediated will be derived from these
  #in this current bootstrap
```

```
  bs.perc.med.array[bs,s,1] <- bs.ie.array[bs,s,1]/te.bs.array[bs,1,1]
  bs.perc.med.array[bs,s,2] <- bs.ie.array[bs,s,2]/te.bs.array[bs,1,2]
  bs.perc.med.array[bs,s,3] <- bs.ie.array[bs,s,3]/te.bs.array[bs,1,3]
  bs.perc.med.array[bs,s,4] <- bs.ie.array[bs,s,4]/te.bs.array[bs,1,4]

}

#Means from nc and cf
#This is to assess simulation fit

for(v in 1:nvar){
  bs.nc.array[bs,v,1] <- mean(nc.array[,v,1])
  bs.nc.array[bs,v,2] <- mean(nc.array[,v,2])
  bs.nc.array[bs,v,3] <- mean(nc.array[,v,3])
  bs.nc.array[bs,v,4] <- mean(nc.array[,v,4])
  bs.nc.array[bs,v,5] <- mean(nc.array[,v,5])
  bs.nc.array[bs,v,6] <- mean(nc.array[,v,6])
  bs.nc.array[bs,v,7] <- mean(nc.array[,v,7])
  bs.nc.array[bs,v,8] <- mean(nc.array[,v,8])
  bs.nc.array[bs,v,9] <- mean(nc.array[,v,9])
  bs.nc.array[bs,v,10] <- mean(nc.array[,v,10])
  bs.nc.array[bs,v,11] <- mean(nc.array[,v,11])
  bs.nc.array[bs,v,12] <- mean(nc.array[,v,12])
  bs.nc.array[bs,v,13] <- mean(nc.array[,v,13])
  bs.nc.array[bs,v,14] <- mean(nc.array[,v,14])
  bs.nc.array[bs,v,15] <- mean(nc.array[,v,15])
  bs.nc.array[bs,v,16] <- mean(nc.array[,v,16])

  bs.cf.array[bs,v,1] <- mean(cf.array[,v,1])
  bs.cf.array[bs,v,2] <- mean(cf.array[,v,2])
  bs.cf.array[bs,v,3] <- mean(cf.array[,v,3])
  bs.cf.array[bs,v,4] <- mean(cf.array[,v,4])
  bs.cf.array[bs,v,5] <- mean(cf.array[,v,5])
  bs.cf.array[bs,v,6] <- mean(cf.array[,v,6])
  bs.cf.array[bs,v,7] <- mean(cf.array[,v,7])
  bs.cf.array[bs,v,8] <- mean(cf.array[,v,8])
  bs.cf.array[bs,v,9] <- mean(cf.array[,v,9])
  bs.cf.array[bs,v,10] <- mean(cf.array[,v,10])
  bs.cf.array[bs,v,11] <- mean(cf.array[,v,11])
  bs.cf.array[bs,v,12] <- mean(cf.array[,v,12])
  bs.cf.array[bs,v,13] <- mean(cf.array[,v,13])
  bs.cf.array[bs,v,14] <- mean(cf.array[,v,14])
  bs.cf.array[bs,v,15] <- mean(cf.array[,v,15])
  bs.cf.array[bs,v,16] <- mean(cf.array[,v,16])

  bs.att.nc.array[bs,v,1] <- mean(att.nc.array[,v,1])
  bs.att.nc.array[bs,v,2] <- mean(att.nc.array[,v,2])


  bs.att.cf.array[bs,v,1] <- mean(att.cf.array[,v,1])
  bs.att.cf.array[bs,v,2] <- mean(att.cf.array[,v,2])
```

```
  }

  print(bs)

}
```

Supplementary File 5: The underlying models for the outcome and mediators used in g-formula simulations.

	Self-harm 18-22			Self-harm 13-17			Psychiatric disorder 13-17		
	OR	95% CI		OR	95% CI		OR	95% CI	
Income									
Highest quintile	ref.			ref.			ref.		
4th quintile	1.10	0.96	1.25	1.06	0.85	1.33	1.01	0.97	1.05
3rd quintile	1.17	1.03	1.33	1.09	0.87	1.37	1.04	1.00	1.08
2nd quintile	1.21	1.06	1.37	1.21	0.97	1.51	1.07	1.03	1.11
Lowest quintile	1.24	1.08	1.41	1.30	1.04	1.63	1.05	1.01	1.10
Parental violent crime									
No	ref.			ref.			ref.		
Yes	1.06	0.93	1.20	1.49	1.19	1.84	1.28	1.22	1.34
Parental substance abuse									
No	ref.			ref.			ref.		
Yes	1.21	1.08	1.35	1.28	1.04	1.56	1.16	1.11	1.21
Parental psychiatric disorder									
No	ref.			ref.			ref.		
Yes	1.26	1.14	1.39	1.87	1.58	2.21	1.97	1.91	2.04
Family stability									
Intact two-parent	ref.			ref.			ref.		
Intact single-parent	1.26	1.14	1.39	1.76	1.46	2.10	1.80	1.74	1.87
Multiple changes	1.38	1.18	1.61	1.85	1.38	2.43	1.74	1.64	1.84
Ever out of family	1.15	0.92	1.43	2.11	1.38	3.09	3.13	2.90	3.37
Disrupted two-parent	1.21	1.07	1.35	1.77	1.44	2.16	1.68	1.62	1.75
Repartnered single parent	1.20	1.04	1.39	1.72	1.32	2.21	1.71	1.63	1.79
Parental education									
Tertiary	ref.			ref.			ref.		
Secondary	1.04	0.96	1.13	1.09	0.95	1.25	1.21	1.18	1.24
Basic	1.04	0.92	1.18	1.39	1.10	1.73	1.55	1.48	1.62
Sex									
Male	ref.			ref.			ref.		
Female	1.53	1.43	1.65	2.86	2.49	3.28	1.40	1.37	1.43
Year of birth									
1995	ref.			ref.			ref.		
1994	1.00	0.88	1.12	0.94	0.77	1.16	0.95	0.91	0.98
1993	1.03	0.92	1.16	0.93	0.76	1.15	0.89	0.85	0.92
1992	1.11	0.98	1.24	0.89	0.72	1.09	0.82	0.79	0.86
1991	1.08	0.96	1.21	0.77	0.62	0.95	0.78	0.75	0.81
1990	1.14	1.02	1.28	0.75	0.61	0.94	0.74	0.71	0.77

NOTE: Mediator ORs for the outcome not shown. Available in the main text

	Substance abuse 13-17			Violent victimization 13-17			Violent crime 13-17		
	OR	95% CI		OR	95% CI		OR	95% CI	
Income									
Highest quintile	ref.			ref.			ref.		
4th quintile	1.04	0.93	1.15	1.00	0.80	1.25	1.26	1.16	1.38
3rd quintile	1.17	1.06	1.30	0.91	0.73	1.15	1.38	1.26	1.50
2nd quintile	1.20	1.08	1.33	0.98	0.78	1.23	1.60	1.47	1.74
Lowest quintile	1.29	1.16	1.44	1.24	1.00	1.56	1.95	1.79	2.12
Parental violent crime									
No	ref.			ref.			ref.		
Yes	1.55	1.41	1.71	1.70	1.38	2.09	2.06	1.93	2.20
Parental substance abuse									
No	ref.			ref.			ref.		
Yes	1.50	1.37	1.64	1.37	1.12	1.68	1.47	1.38	1.57
Parental psychiatric disorder									
No	ref.			ref.			ref.		
Yes	1.36	1.25	1.48	1.14	0.94	1.38	1.10	1.03	1.17
Family stability									
Intact two-parent	ref.			ref.			ref.		
Intact single-parent	1.87	1.72	2.03	1.57	1.30	1.88	1.61	1.51	1.71
Multiple changes	2.00	1.76	2.27	2.34	1.80	3.01	1.90	1.73	2.09
Ever out of family	1.98	1.63	2.38	2.25	1.52	3.22	2.09	1.83	2.37
Disrupted two-parent	1.64	1.49	1.81	1.49	1.19	1.85	1.57	1.46	1.69
Repartnered single parent	1.96	1.75	2.18	1.95	1.52	2.47	1.83	1.68	1.99
Parental education									
Tertiary	ref.			ref.			ref.		
Secondary	1.24	1.17	1.33	1.32	1.15	1.53	1.76	1.67	1.85
Basic	1.74	1.57	1.92	2.21	1.78	2.72	3.08	2.87	3.30
Sex									
Male	ref.			ref.			ref.		
Female	1.10	1.04	1.16	0.40	0.35	0.46	0.32	0.30	0.33
Year of birth									
1995	ref.			ref.			ref.		
1994	0.93	0.85	1.03	1.05	0.86	1.28	1.04	0.96	1.11
1993	0.89	0.81	0.98	0.77	0.62	0.96	0.95	0.88	1.02
1992	0.80	0.73	0.89	0.81	0.66	1.00	0.90	0.84	0.97
1991	0.85	0.77	0.94	0.76	0.62	0.95	0.89	0.83	0.96
1990	0.81	0.74	0.89	0.71	0.57	0.88	0.85	0.79	0.91

	Placement 13-17			NEET 13-17			Lowest GPA quartile		
	OR	95% CI		OR	95% CI		OR	95% CI	
Income									
Highest quintile	ref.			ref.			ref.		
4th quintile	1.30	1.17	1.44	0.98	0.93	1.03	1.44	1.40	1.48
3rd quintile	1.68	1.53	1.86	1.14	1.08	1.19	1.72	1.67	1.77
2nd quintile	2.22	2.03	2.44	1.39	1.33	1.45	1.95	1.90	2.01
Lowest quintile	2.91	2.66	3.20	1.81	1.73	1.90	2.19	2.13	2.25
Parental violent crime									
No	ref.			ref.			ref.		
Yes	1.83	1.73	1.94	1.34	1.28	1.40	1.34	1.29	1.39
Parental substance abuse									
No	ref.			ref.			ref.		
Yes	1.78	1.69	1.88	1.14	1.09	1.19	1.32	1.28	1.37
Parental psychiatric disorder									
No	ref.			ref.			ref.		
Yes	1.90	1.81	2.00	1.23	1.18	1.28	1.03	1.00	1.06
Family stability									
Intact two-parent	ref.			ref.			ref.		
Intact single-parent	2.52	2.38	2.66	1.51	1.46	1.57	1.26	1.23	1.29
Multiple changes	2.99	2.76	3.24	1.53	1.44	1.62	1.52	1.46	1.58
Ever out of family	7.11	6.48	7.78	2.43	2.24	2.63	1.58	1.48	1.69
Disrupted two-parent	2.49	2.34	2.66	1.43	1.37	1.50	1.37	1.33	1.40
Repartnered single parent	2.43	2.25	2.63	1.50	1.43	1.59	1.43	1.38	1.48
Parental education									
Tertiary	ref.			ref.			ref.		
Secondary	1.84	1.75	1.93	1.71	1.66	1.76	2.48	2.44	2.52
Basic	3.15	2.95	3.36	3.37	3.24	3.52	4.04	3.92	4.17
Sex									
Male	ref.			ref.			ref.		
Female	1.19	1.14	1.24	1.03	1.01	1.06	0.38	0.37	0.38
Year of birth									
1995	ref.			ref.			ref.		
1994	0.93	0.87	0.99	1.03	0.99	1.08	1.00	0.98	1.03
1993	0.78	0.73	0.83	1.04	1.00	1.09	0.93	0.90	0.95
1992	0.71	0.66	0.76	1.05	1.00	1.10	0.96	0.94	0.99
1991	0.69	0.64	0.73	1.13	1.08	1.17	0.94	0.92	0.97
1990	0.62	0.58	0.66	0.95	0.91	1.00	0.99	0.96	1.01

	2nd GPA quartile			3rd GPA quartile			Highest GPA quartile		
	OR	95% CI		OR	95% CI		OR	95% CI	
Income									
Highest quintile	ref.			ref.			ref.		
4th quintile	1.29	1.25	1.32	1.00	0.97	1.02	0.67	0.66	0.69
3rd quintile	1.39	1.35	1.42	0.95	0.93	0.97	0.56	0.54	0.57
2nd quintile	1.42	1.38	1.45	0.87	0.85	0.89	0.50	0.49	0.52
Lowest quintile	1.38	1.34	1.42	0.81	0.79	0.83	0.45	0.44	0.46
Parental violent crime									
No	ref.			ref.			ref.		
Yes	1.00	0.97	1.04	0.83	0.80	0.87	0.68	0.65	0.72
Parental substance abuse									
No	ref.			ref.			ref.		
Yes	0.99	0.96	1.02	0.87	0.83	0.90	0.71	0.68	0.74
Parental psychiatric disorder									
No	ref.			ref.			ref.		
Yes	1.04	1.01	1.07	0.95	0.92	0.98	0.97	0.94	1.00
Family stability									
Intact two-parent	ref.			ref.			ref.		
Intact single-parent	0.99	0.96	1.01	0.91	0.88	0.93	0.86	0.83	0.88
Multiple changes	1.06	1.01	1.11	0.84	0.80	0.88	0.61	0.58	0.65
Ever out of family	0.85	0.79	0.91	0.77	0.71	0.84	0.82	0.75	0.89
Disrupted two-parent	1.05	1.02	1.08	0.88	0.86	0.91	0.74	0.72	0.77
Repartnered single parent	1.05	1.01	1.09	0.89	0.85	0.92	0.66	0.63	0.70
Parental education									
Tertiary	ref.			ref.			ref.		
Secondary	1.27	1.25	1.29	0.73	0.72	0.74	0.39	0.38	0.39
Basic	1.12	1.08	1.16	0.52	0.50	0.54	0.23	0.21	0.24
Sex									
Male	ref.			ref.			ref.		
Female	0.81	0.80	0.83	1.30	1.28	1.32	2.72	2.67	2.76
Year of birth									
1995	ref.			ref.			ref.		
1994	0.95	0.93	0.98	1.01	0.98	1.04	1.03	1.01	1.06
1993	0.96	0.93	0.98	1.01	0.99	1.04	1.11	1.08	1.14
1992	0.93	0.91	0.95	1.01	0.98	1.03	1.12	1.09	1.15
1991	0.92	0.90	0.94	1.03	1.00	1.05	1.13	1.10	1.17
1990	0.85	0.83	0.87	1.08	1.06	1.11	1.10	1.07	1.14

Supplementary file 6: Natural course means and observed means of the outcome and mediators

	Natural course average (NC)	95 % CI		Observed aveage value	Ratio of observed and NC average values
Self-harm 18-22	0.008	0.008	0.008	0.009	1.14
Self-harm 13-17	0.003	0.003	0.003	0.003	1.00
Psychiatric disorder	0.087	0.087	0.088	0.087	1.00
Substance abuse	0.013	0.013	0.013	0.013	1.00
Violent victimization	0.003	0.002	0.003	0.003	1.00
Violent crime	0.025	0.024	0.025	0.025	1.00
NEET	0.075	0.074	0.076	0.075	1.00
Out-of-home placement	0.028	0.028	0.029	0.028	1.00
Lowest GPA quartile	0.274	0.272	0.275	0.274	1.00
2nd GPA quartile	0.244	0.242	0.245	0.243	1.00
3rd GPA quartile	0.250	0.249	0.252	0.249	1.00
Highest GPA quartile	0.233	0.231	0.234	0.234	1.00

One way to assess that the mediators and outcome are predicted correctly in the g-formula is to compare the natural course (NC) mean (averaged over bootstrap iterations) to the expected values of the variables in the observational data. Both of these estimates can be interpreted as the proportion of individuals with a given outcome. The comparison is done as a check against gross model misspecification. The rationale behind this is that the predictions in the natural course are done without intervening in the data and thus reflect the real world scenario. If the natural course predictions are not close to observed average values, then models for outcome and/or mediators are likely to be incorrectly specified.

Above we can see that the models for mediators predict natural course means very close to the observed values. The predictions with the model for outcome, self-harm at age 18–22, slightly underestimate the proportion of individuals with self-harm. This relates to the fact that in the case of mediators, only observed values are used for predictions but in the case of the outcome, the mediators are first predicted and then these values used to predict the outcome. Thus, there is more room for error. Another explanation for the underestimation might be that logistic regression can underestimate rare events. When comparing absolute levels, this may cause problems, which can be partly alleviated with the use of relative numbers (King and Zeng 2001).

King, G., Zeng, L., 2001. Logistic Regression in Rare Events Data. Polit. Anal. 9, 137–163. https://doi.org/10.1093/oxfordjournals.pan.a004868

Supplementary file 7: Absolute effect sizes among the total population and the treated

	Effect size	95% CI	
Total Effect (TE)			
TE, on self-harm 18-22	-0.000173	-0.000411	-0.000004
TE, on self-harm 13-17	-0.000064	-0.000190	0.000056
TE, on psychiatric disorder 13-17	0.000266	-0.000410	0.000943
TE, on substance abuse 13-17	-0.000278	-0.000560	-0.000018
TE, on violent victimization 13-17	-0.000182	-0.000324	-0.000041
TE, on violent crime 13-17	-0.001500	-0.001917	-0.001054
TE, on NEET 13-17	-0.005442	-0.006198	-0.004698
TE, on placements 13-17	-0.002728	-0.003253	-0.002269
TE, on lowest GPA quartile	-0.004964	-0.005911	-0.004073
TE, on second GPA quartile	0.000784	0.000001	0.001747
TE, on third GPA quartile	0.001901	0.001108	0.002688
TE, on highest GPA quartile	0.002279	0.001638	0.002885
Average Treatment effect for the treated (ATT)			
ATT, on self-harm 18-22	-0.000853	-0.002030	-0.000001
ATT, on self-harm 13-17	-0.000320	-0.000933	0.000272
ATT, on psychiatric disorder 13-17	0.001329	-0.002173	0.004634
ATT, on substance abuse 13-17	-0.001390	-0.002818	-0.000220
ATT, on violent victimization 13-17	-0.000906	-0.001581	-0.000202
ATT, on violent crime 13-17	-0.007494	-0.009565	-0.005361
ATT, on NEET 13-17	-0.027198	-0.031126	-0.023480
ATT, on placements 13-17	-0.013626	-0.016158	-0.011338
ATT, on lowest GPA quartile	-0.024832	-0.029645	-0.020490
ATT, on second GPA quartile	0.003910	0.000127	0.008220
ATT, on third GPA quartile	0.009563	0.005205	0.013721
ATT, on highest GPA quartile	0.011359	0.008218	0.014418

	Effect size	95% CI	
Natural indirect effect (NIE) in total population			
Scenario 1	-0.000116	-0.000163	-0.000073
Scenario 2	-0.000122	-0.000161	-0.000074
Scenario 3	-0.000113	-0.000162	-0.000066
Scenario 4	-0.000112	-0.000156	-0.000065
Scenario 5	-0.000101	-0.000143	-0.000054
Scenario 6	-0.000114	-0.000159	-0.000067
Scenario 7	-0.000092	-0.000137	-0.000049
Scenario 8	-0.000086	-0.000132	-0.000039
Scenario 9	-0.000068	-0.000112	-0.000024
Total direct effect (TDE) in total population			
Scenario 1	-0.000056	-0.000295	0.000131
Scenario 2	-0.000051	-0.000298	0.000144
Scenario 3	-0.000060	-0.000296	0.000126
Scenario 4	-0.000060	-0.000299	0.000123
Scenario 5	-0.000072	-0.000306	0.000109
Scenario 6	-0.000059	-0.000294	0.000132
Scenario 7	-0.000081	-0.000330	0.000113
Scenario 8	-0.000087	-0.000326	0.000110
Scenario 9	-0.000105	-0.000336	0.000069
Natural indirect effect (NIE) among treated			
Scenario 1	-0.000567	-0.000747	-0.000386
Scenario 2	-0.000600	-0.000730	-0.000454
Scenario 3	-0.000554	-0.000701	-0.000381
Scenario 4	-0.000552	-0.000711	-0.000389
Scenario 5	-0.000495	-0.000664	-0.000331
Scenario 6	-0.000563	-0.000715	-0.000389
Scenario 7	-0.000452	-0.000615	-0.000285
Scenario 8	-0.000419	-0.000583	-0.000252
Scenario 9	-0.000334	-0.000500	-0.000178
Total direct effect (TDE) among treated			
Scenario 1	-0.000286	-0.001456	0.000656
Scenario 2	-0.000253	-0.001469	0.000672
Scenario 3	-0.000300	-0.001487	0.000617
Scenario 4	-0.000301	-0.001455	0.000631
Scenario 5	-0.000358	-0.001516	0.000568
Scenario 6	-0.000290	-0.001481	0.000616
Scenario 7	-0.000401	-0.001593	0.000529
Scenario 8	-0.000434	-0.001623	0.000483
Scenario 9	-0.000520	-0.001666	0.000375

Scenario 1: All mediators fixed to Natural course values
Scenarios 2-9: Psychiatric disorders (2), substance abuse (3), previous self-harm (4), violent crime (5), victimization (6), NEET (7), GPA (8) and out-of-home placement (9) fixed to counterfactual values.

Supplementary file 8: Evaluating rare outcomes

Rare outcomes in mediation analysis

Regarding mediation analysis with rare events and the challenges involved on a general level, we are unaware of other mediation methods that are better suited at modeling rare events than the g-formula. Previous work does indicate that with rare binary outcomes, the non-collapsibility related issues are avoided (VanderWeele, 2016). Thus, rare outcome in itself should not pose big problems for mediation analysis, and is even a desirable property with binary outcomes. However, the rarity of outcome is conditional on all the combinations of exposure and mediator (Samoilenko and Lefebvre, 2019; VanderWeele et al., 2019). In our case, self-harm at age 18-22 is actually common among those with self-harm at age 13-17 (17%). Therefore, potential biases related to non-collapsible estimates may result. The g-formula, even though a rather heavy choice of a method, does solve for these problems by relying on standardization. In addition, the flexibility of the g-formula allows for studying custom interventions, which can be helpful in many applications.

Turning to logistic regression modelling and the rare outcomes, King & Zeng (2001) demonstrate that logistic models might underestimate rare outcomes. However, relative rareness of the outcome does not necessarily pose big problems, if the sample size is sufficiently large and thus there are enough cases in absolute numbers (see Paul Allison's blog post on the issue here: https://statisticalhorizons.com/logistic-regression-for-rare-events). As we have around 384000 individuals with around 3,600 events of self-harm, the bias should be small. Indeed, in our Supplementary file 6 on the correct predictions, the logistic model does seem to work sufficiently well. This is also demonstrated in the simulations below

Allison, P., 2012. Logistic Regression for Rare Events [WWW Document]. URL https://statisticalhorizons.com/logistic-regression-for-rare-events (accessed 1.15.21).

King, G., Zeng, L., 2001. Logistic Regression in Rare Events Data. Polit. Anal. 9, 137–163. https://doi.org/10.1093/oxfordjournals.pan.a004868

Samoilenko, M., Lefebvre, G., 2019. Point: Risk Ratio Equations for Natural Direct and Indirect Effects in Causal Mediation Analysis of a Binary Mediator and a Binary Outcome—A Fresh Look at the Formulas. Am. J. Epidemiol. 188, 1201–1203. https://doi.org/10.1093/aje/kwy275

VanderWeele, T.J., 2016. Mediation Analysis: A Practitioner's Guide. Annu. Rev. Public Health 37, 17–32. https://doi.org/10.1146/annurev-publhealth-032315-021402

VanderWeele, T.J., Valeri, L., Ananth, C.V., 2019. Counterpoint: Mediation Formulas With Binary Mediators and Outcomes and the "Rare Outcome Assumption." Am. J. Epidemiol. 188, 1204–1205. https://doi.org/10.1093/aje/kwy281

Effect Distributions

Below we plot the density plots of all the effects, as produced by the 250 bootstrap iterations. As can be seen from the plots, the effects are approximately normally distributed. Another observation is that the Total Direct Effect is centered around 0, further confirming the result of high amount of mediation of the total effect. Furthermore, the indirect effect has far less variation than total and direct effect, which is due to the calculation process: the total direct effect is heavily correlated with total effect size and thus the difference between these two effects has less variation. Lastly, the tail of the Total Effect is slightly positive, which we already note in the manuscript: the upper limit of 95% confidence interval was practically 0.

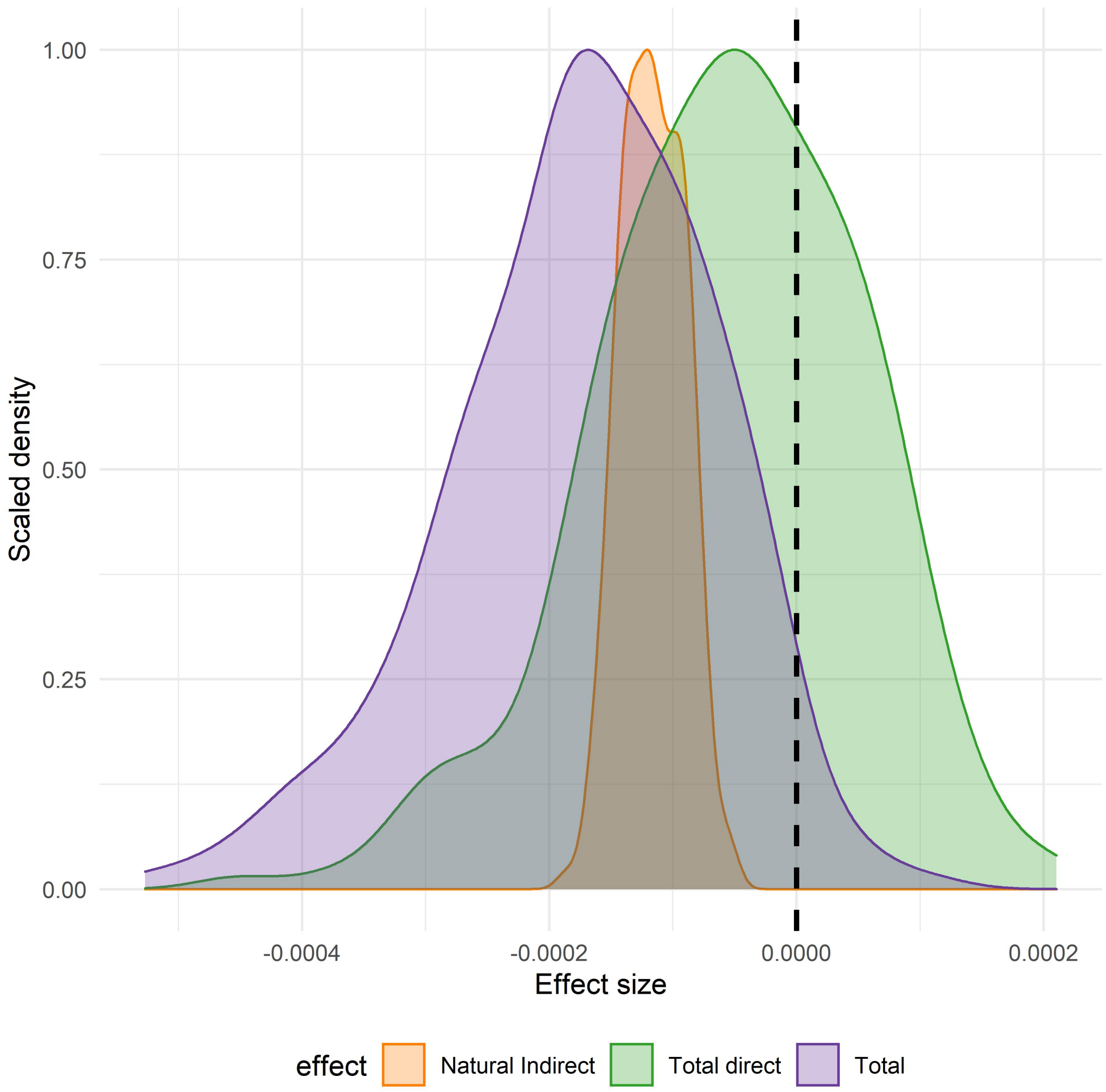

Unstable estimates

We refer to unstable estimates due to rare events in the manuscript. It is important to note that this does not seem to impact much the estimation of the absolute effects. However, the calculation of the percentage mediated, which is a ratio, is affected by the rareness of the outcome. Values near zero may easily blow up the ratio. Moreover, as there are some iterations were TE is quite small but above 0 (6 iterations), and iterations were TDE is positive and indirect negative (82 in total, the 6 iterations where TE was positive included), the calculation of the average ratio was not informative. We did a check where we truncated the observations of percentage mediated to exclude all the values where percentage mediated was below 0% and over 100%. After truncation, we were left with 168 bootstrap iterations, with mean percentage mediated of 55% (95% CI: 0.24,0.93). This is not very far from our estimation of 67% in the manuscript, but the confidence intervals are much wider than those calculated by hand (normal approximation). However, it still is safe to say that most of the effect of income on self-harm is indirect, even if the 67% estimate we calculated would be wrong. Most importantly, as the indirect effect is statistically significant (all iterations under 0), we can infer that targeting groups that are exposed to the mediators is a good way to prevent self-harm related to low income.

Simulation study

To further investigate whether the issues related to rare outcomes would impact our results on the effect

estimates, we conducted a small-scale simulation study. We first generate artificial data with a sample size of 384,000 (derived from our main study), a binary exposure that has a prevalence of 30%, two binary mediators with prevalence around 30% and 12%, and a considerably rare outcome (prevalence 1%). We run the g-formula for this dataset and calculate Total Effect (TE), Total Direct Effect (TDE) and Natural Indirect Effect (NIE). Our intervention in the g-formula is to set the exposure to 0. As in the main paper, we use logistic regression to model the associations between exposure, mediators and outcome. We do the data generation process and g-formula effect estimation 500 times. Inside the data generation process loop, we stabilize the effect estimates by calculating natural course, counterfactual and mediation scenario averages for each variable 60 times.

After the effect estimation, we calculate true effects. This is done by first generating a natural course scenario, where we create a data with the same data generation mechanism as the data created for the g-formula. Then we copy this data, set exposure to 0, and create a counterfactual scenario, again with the same parameters. Lastly we create a mediation scenario where we set exposure to 0 and draw mediator values from the natural course scenario. We calculate same effects as in the g-formula step. All the scenario simulations are repeated 60 times to reduce Monte Carlo error. The effects derived can be referred as true effects since we know the actual mechanism that creates the data.

Results

The results of the simulations are presented below. The 500 effects produced with the g-formula neatly center around the true effect, and this applies to all the estimated effects. Taking the average of the 500 effect estimates and deducting the true effect from these produced numbers practically 0 for each of the effects. Therefore, the g-formula does seem to perform well, even if we have a rare outcome and model it with logistic regression. We also demonstrate below that the natural indirect effect has more narrow distribution than total and total direct effect, which is similar to our main findings, and related to the calculation of indirect effect as the difference between total effect and total direct effect.

Then, we looked at the percentage mediated causing trouble in our main study. The true percentage mediated was 5.6% in the simulation study. The g-formula gives an estimate of 5.7% for the percentage mediated, when calculated as the average of the ratios of NIE to TE. When calculated as the ratio of average NIE to average TE, the result is practically the same, 5.7%, with some differences in decimals. Therefore, at least with these estimates, the calculation of percentage mediated as a ratio of two averages seems to yield approximately correct results. Taken from the distribution of the 500 ratios, the 2.5th quintile is 3.2%, 97.5th 8.4%. Calculated based on normal approximation the CI for the ratio of the two averages was 5.6% and 5.8%. This might indicate that our confidence intervals in the main paper could be too narrow. However, these cannot be directly compared as we do not bootstrap in the simulation study. Furthermore, based on the density plot above, we can safely say that the indirect effect in our main study is nevertheless statistically significant.

Lastly, to evaluate whether the logistic model used in the g-formula underestimates the outcome, we also compared the average of the outcome in each of the 500 generated datasets to their Monte Carlo stabilized natural course counterparts. The mean difference between these two estimates was practically 0 (2.5th and 97.5th percentiles -0.000004,0.000005), indicating that the logistic model does work sufficiently well with large sample size, even though the outcome is considerably rare in relative terms.

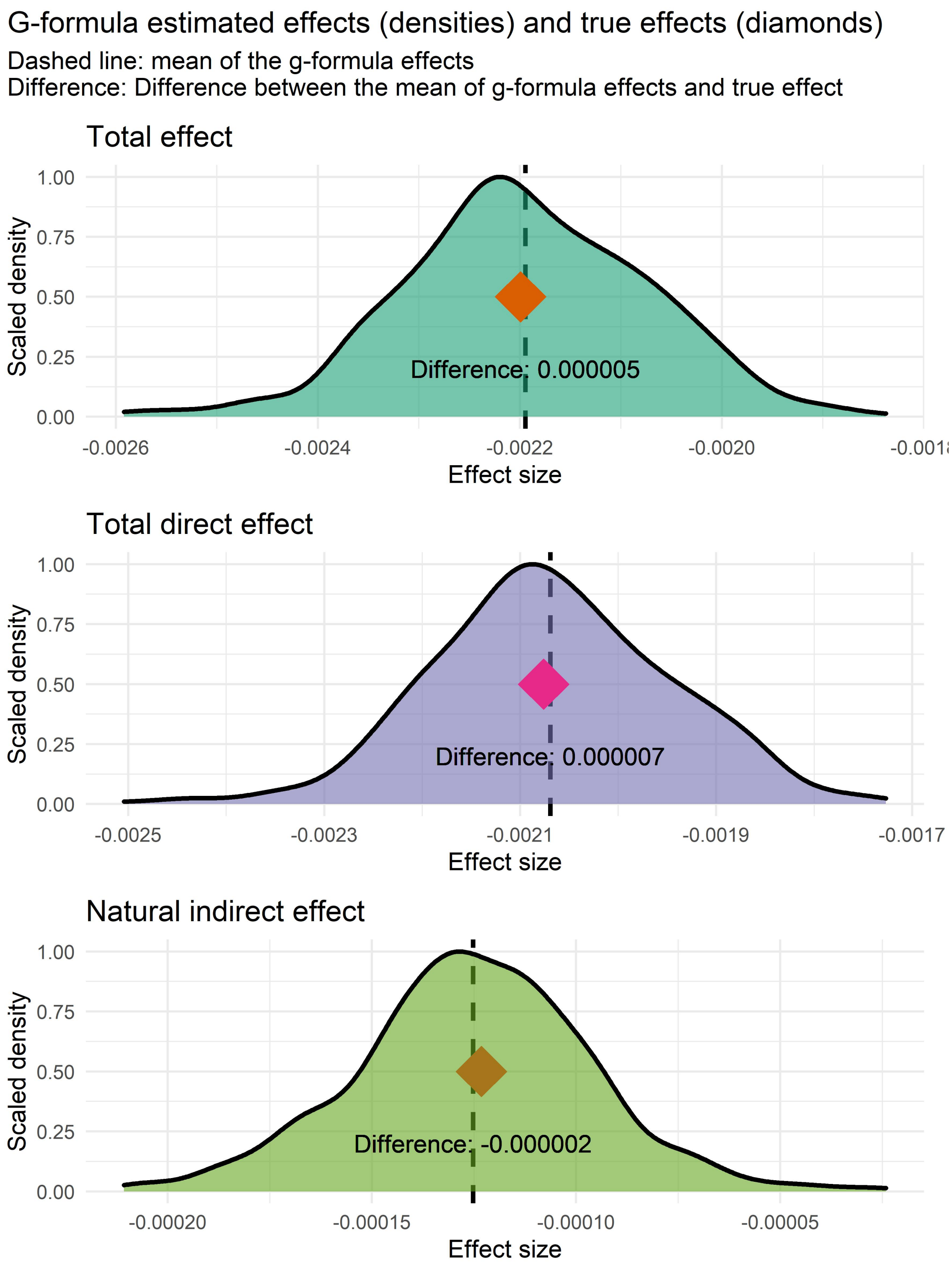
G-formula estimated effects (densities) and true effects (diamonds)
Dashed line: mean of the g-formula effects
Difference: Difference between the mean of g-formula effects and true effect
Total effect
Scaled density
1.00
0.75
0.50
0.25
0.00
Difference: 0.000005
-0.0026
-0.0024
-0.0022
-0.0020
Effect size
Total direct effect
Scaled density
1.00
0.75
0.50
0.25
0.00
Difference: 0.000007
-0.0025
-0.0023
-0.0021
-0.0019
-0.0017
Effect size
Natural indirect effect
Scaled density
1.00
0.75
0.50
0.25
0.00
Difference: -0.000002
-0.00020
-0.00015
-0.00010
-0.00005
Effect size

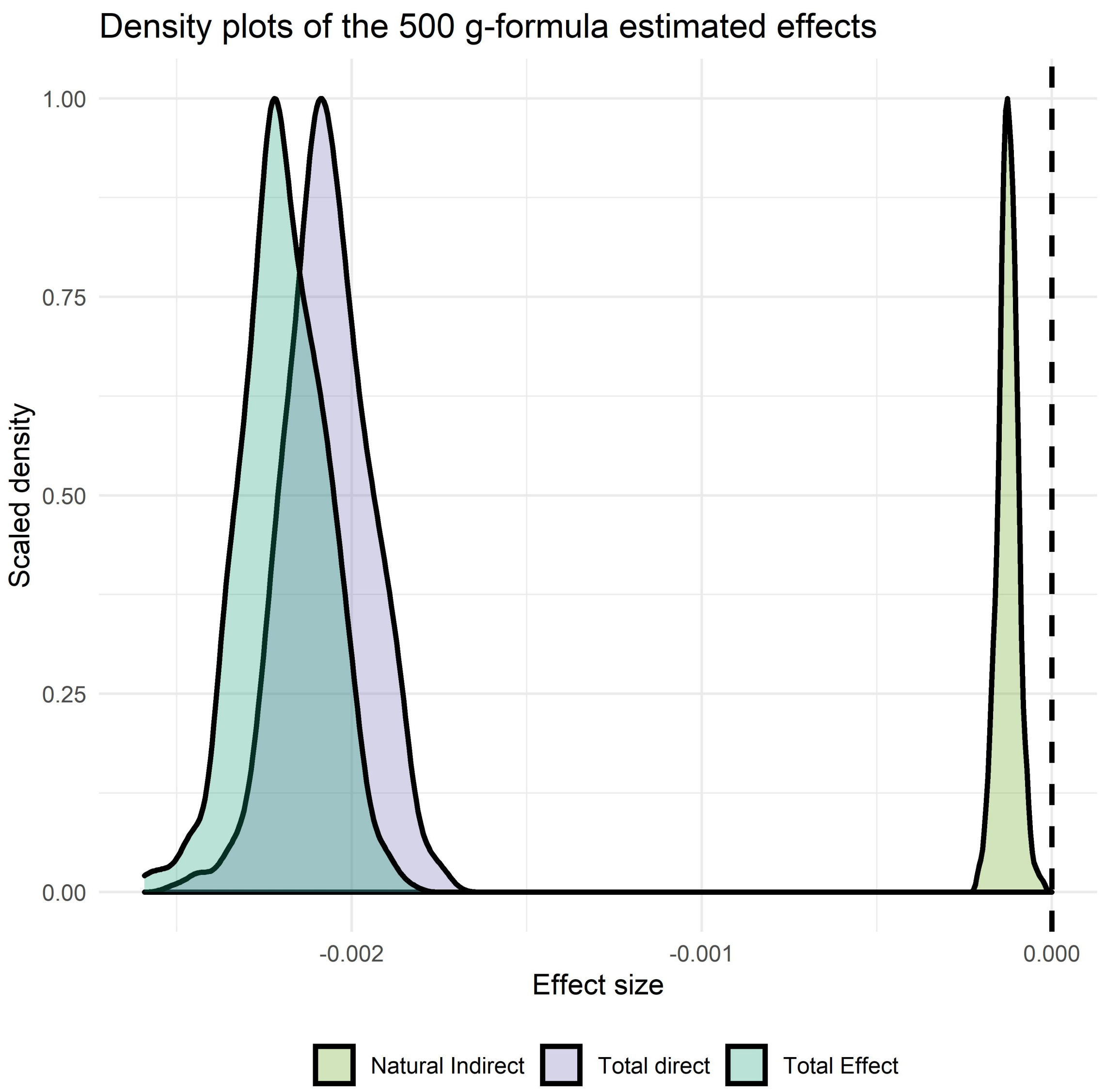
Density plots of the 500 g-formula estimated effects
Scaled density
1.00
0.75
0.50
0.25
0.00
-0.002
-0.001
0.000
Effect size
Natural Indirect
Total direct
Total Effect

Code for the simulation study

```
#Simulation study to check
#sensitivity for rare outcome

#Expit function
expit <- function(x) {exp(x)/(1+exp(x))}

#Create data

n <- 384000 #sample size close to the one in the actual paper
X <- rbinom(n,1,expit(-1+0.2)) # binary exposure with prevalence around 30%
M1 <- rbinom(n,1,expit(-2+0.1*X)) #binary mediator, around 12% prevalence
M2 <- rbinom(n,1,expit(-1+0.5*X)) #binary mediator, around 30% prevalence
Y <- rbinom(n,1,expit(-5+0.6*X+M1*0.3+M2*0.4)) # Outcome, prevalence around 1%

dataset <- as.data.frame(cbind(X,M1,M2,Y))

#First, estimate how many Monte Carlo loops
#are needed in g-formula
#(when does the estimate stabilize)

mtest <- 100
mtest.mat <- matrix(NA,nrow=100,ncol=3)
#Fit models:

model_mediator_1 <- glm(M1 ~ X, family='binomial', data=dataset)
model_mediator_2 <- glm(M2 ~ X, family='binomial', data=dataset)
model_outcome <- glm(Y ~ X + M1 + M2, family='binomial', data=dataset)

for (m in 1:mtest){

  dataset2 <- dataset

  dataset2$M1 <- rbinom(n,1,expit(
    predict(object=model_mediator_1,newdata=dataset2)))
  dataset2$M2 <- rbinom(n,1,expit(
    predict(object=model_mediator_2,newdata=dataset2)))

  #Lastly, predict new vaues for the outcome

  dataset2$Y <- rbinom(n,1,expit(
    predict(object=model_outcome,newdata=dataset2)))

  mtest.mat[m,1] <- mean(dataset2$Y)
  mtest.mat[m,2] <- mean(dataset2$M1)
  mtest.mat[m,3] <- mean(dataset2$M2)

  print(m)

}

plot.y.vec <- c(rep(NA,100))
plot.m1.vec <- c(rep(NA,100))
```

```r
plot.m2.vec <- c(rep(NA,100))

for(i in 1:100){
  plot.y.vec[i] <- mean(mtest.mat[1:i,1])
  plot.m1.vec[i] <- mean(mtest.mat[1:i,2])
  plot.m2.vec[i] <- mean(mtest.mat[1:i,3])

  print(i)

}

plot(plot.y.vec, type='l') #around 60
plot(plot.m1.vec, type='l') #around 60
plot(plot.m2.vec, type='l') #around 40

#60 seems a safe option

########################
#G-FORMULA PREPARATIONS#
########################

msize <- 60
dgpsize <- 500

#Monte Carlo matrices for means
cf.mat <- nc.mat <- matrix(NA,nrow=msize, ncol=3)
med.mat <- matrix(NA,nrow=msize, ncol=1)

#Effect matrix for 500 total effects

#all vars
effect.mat <- matrix(NA,nrow=dgpsize,ncol=3)

#Matrix for direct, indirect and % mediated
direct.mat <- matrix(NA,nrow=dgpsize,ncol=3)

#Mean matrix for bootstraps
#NC, randomly created data and constant probabilities of outcome

comparison.mat <- matrix(NA,nrow=dgpsize,ncol=2)

#################
#START G-FORMULA#
#################

#Data generation process

for (bs in 1:dgpsize){

  n <- 384000
  X <- rbinom(n,1,expit(-1+0.2))
  M1 <- rbinom(n,1,expit(-2+0.1*X))
  M2 <- rbinom(n,1,expit(-1+0.5*X))
```

```r
Y <- rbinom(n,1,expit(-5+0.6*X+M1*0.3+M2*0.4))

dataset <- as.data.frame(cbind(X,M1,M2,Y))

model_mediator_1 <- glm(M1 ~ X, family='binomial', data=dataset)
model_mediator_2 <- glm(M2 ~ X, family='binomial', data=dataset)
model_outcome <- glm(Y ~ X + M1 + M2, family='binomial', data=dataset)

#Monte Carlo loops to reduce MC error

for (m in 1:msize) {

  #Natural course step:

  #Copy data
  sample.xMx <- dataset

  #Replace mediator values with newly predicted values

  sample.xMx$M1 <- rbinom(n,1,expit(
    predict(object=model_mediator_1,newdata=sample.xMx)))
  sample.xMx$M2 <- rbinom(n,1,expit(
    predict(object=model_mediator_2,newdata=sample.xMx)))

  #Lastly, predict new values for the outcome

  sample.xMx$Y <- rbinom(n,1,expit(
    predict(object=model_outcome,newdata=sample.xMx)))

  #####################
  #Counterfactual step#
  #####################

  #Copy the sampled data

  sample.xstarMxstar <- dataset

  #Make an intervention
  #Here, remove X

  sample.xstarMxstar$X <- 0


  #Replace mediator values with newly predicted values

  sample.xstarMxstar$M1 <- rbinom(n,1,expit(
    predict(object=model_mediator_1,newdata=sample.xstarMxstar)))
  sample.xstarMxstar$M2 <- rbinom(n,1,expit(
    predict(object=model_mediator_2,newdata=sample.xstarMxstar)))

  #Lastly, predict new vaues for the outcome

  sample.xstarMxstar$Y <- rbinom(n,1,expit(
```

```
    predict(object=model_outcome,newdata=sample.xstarMxstar)))

  #####################
  #Total direct effect#
  #####################

  #Copy data
  sample.xstarMx <- dataset


  #Make an intervention
  #Here, remove X

  sample.xstarMx$X <- 0


  #Replace mediator values with vaölues from Natural course

  sample.xstarMx$M1 <- sample.xMx$M1
  sample.xstarMx$M2 <- sample.xMx$M2

  #Lastly, predict new vaues for the outcome

  sample.xstarMx$Y <- rbinom(n,1,expit(
    predict(object=model_outcome,newdata=sample.xstarMx)))

  nc.mat[m,1] <- mean(sample.xMx$Y)
  nc.mat[m,2] <- mean(sample.xMx$M1)
  nc.mat[m,3] <- mean(sample.xMx$M2)


  cf.mat[m,1] <- mean(sample.xstarMxstar$Y)
  cf.mat[m,2] <- mean(sample.xstarMxstar$M1)
  cf.mat[m,3] <- mean(sample.xstarMxstar$M2)

  #Mediation

  med.mat[m,1] <- mean(sample.xstarMx$Y)

}


#After all the MC iterations,
#save the bootstrap results


#Calculate effects

##############
#Total effect#
##############

effect.mat[bs,1] <- mean(cf.mat[,1])-mean(nc.mat[,1]) #Y
```

```
  effect.mat[bs,2] <- mean(cf.mat[,2])-mean(nc.mat[,2]) #M1
  effect.mat[bs,3] <- mean(cf.mat[,3])-mean(nc.mat[,3]) #M2

  ###########
  #Mediation#
  ###########

  direct.mat[bs,1] <- mean(med.mat[,1])-mean(nc.mat[,1]) #direct effect
  direct.mat[bs,2] <- effect.mat[bs,1]-direct.mat[bs,1] #indirect effect
  direct.mat[bs,3] <- direct.mat[bs,2]/effect.mat[bs,1] #% mediated


  ############
  #Comparison#
  ############

  #NC and actual data creation

  comparison.mat[bs,1] <- mean(nc.mat[,1]) - mean(dataset$Y)
  comparison.mat[bs,2] <- mean(nc.mat[,1]) - mean(expit(-5+0.6*X+M1*0.3+M2*0.4))

  print(bs)

}

###############
#END G-FORMULA#
###############

#Calculate true Total, Direct and Indirect Effect

#Test how many MC loops are needed

m1.nc.vec <- c(rep(NA),mtest)
m2.nc.vec <- c(rep(NA),mtest)
y.nc.vec <- c(rep(NA),mtest)

m1.cf.vec <- c(rep(NA),mtest)
m2.cf.vec <- c(rep(NA),mtest)
y.cf.vec <- c(rep(NA),mtest)


for (m in 1:mtest){

  nc.data <-
    cf.data <-
    dataset

  cf.data$X <- 0

  n <- 384000
  M1.nc <- rbinom(n,1,expit(-2+0.1*nc.data$X))
  M2.nc <- rbinom(n,1,expit(-1+0.5*nc.data$X))
```

```
  Y.nc <- expit(-5+0.6*nc.data$X+nc.data$M1*0.3+nc.data$M2*0.4)

  M1.cf <- rbinom(n,1,expit(-2+0.1*cf.data$X))
  M2.cf <- rbinom(n,1,expit(-1+0.5*cf.data$X))
  Y.cf <- expit(-5+0.6*cf.data$X+cf.data$M1*0.3+cf.data$M2*0.4)

  m1.nc.vec[m] <- mean(M1.nc)
  m2.nc.vec[m] <- mean(M2.nc)
  y.nc.vec[m] <- mean(Y.nc)

  m1.cf.vec[m] <- mean(M1.cf)
  m2.cf.vec[m] <- mean(M2.cf)
  y.cf.vec[m] <- mean(Y.cf)

  print(m)


}

plot.m1.nc.vec <- c(rep(NA,mtest))
plot.m2.nc.vec <- c(rep(NA,mtest))
plot.y.nc.vec <- c(rep(NA,mtest))

plot.m1.cf.vec <- c(rep(NA,mtest))
plot.m2.cf.vec <- c(rep(NA,mtest))
plot.y.cf.vec <- c(rep(NA,mtest))

for(i in 1:100){

  plot.m1.nc.vec[i] <- mean(m1.nc.vec[1:i])
  plot.m1.cf.vec[i] <- mean(m1.cf.vec[1:i])

  plot.m2.nc.vec[i] <- mean(m2.nc.vec[1:i])
  plot.m2.cf.vec[i] <- mean(m2.cf.vec[1:i])

  plot.y.nc.vec[i] <- mean(y.nc.vec[1:i])
  plot.y.cf.vec[i] <- mean(y.cf.vec[1:i])


  print(i)

}

plot(plot.m1.nc.vec, type='l') #around 60
plot(plot.m1.cf.vec, type='l') #around 60

plot(plot.m2.nc.vec, type='l') #around 60
plot(plot.m2.cf.vec, type='l') #around 60

plot(plot.y.vec, type='l') #around 60
plot(plot.y.vec, type='l') #around 60

#Simulate data and calculate effects
```

```
msize <- 60

m1.nc.vec <- c(rep(NA),msize)
m2.nc.vec <- c(rep(NA),msize)
y.nc.vec <- c(rep(NA),msize)

m1.cf.vec <- c(rep(NA),msize)
m2.cf.vec <- c(rep(NA),msize)
y.cf.vec <- c(rep(NA),msize)

y.med.vec <- c(rep(NA),msize)

effects.y <- matrix(NA,msize,4)
effects.m1 <- c(rep(NA),msize)
effects.m2 <- c(rep(NA),msize)

for (m in 1:msize){

  nc.data <-
    cf.data <-
    mediation <-
    dataset

  cf.data$X <- 0
  mediation$X <- 0

  n <- 384000 #sample size close to the one in rge actual paper
  nc.data$M1 <- rbinom(n,1,expit(-2+0.1*nc.data$X))
  nc.data$M2 <- rbinom(n,1,expit(-1+0.5*nc.data$X))
  nc.data$Y <- expit(-5+0.6*nc.data$X+nc.data$M1*0.3+nc.data$M2*0.4)

  cf.data$M1 <- rbinom(n,1,expit(-2+0.1*cf.data$X))
  cf.data$M2 <- rbinom(n,1,expit(-1+0.5*cf.data$X))
  cf.data$Y <- expit(-5+0.6*cf.data$X+cf.data$M1*0.3+cf.data$M2*0.4)

  mediation$M1 <- nc.data$M1
  mediation$M2 <- nc.data$M2
  mediation$Y <- expit(-5+0.6*mediation$X+mediation$M1*0.3+mediation$M2*0.4)

  m1.nc.vec[m] <- mean(nc.data$M1)
  m2.nc.vec[m] <- mean(nc.data$M1)
  y.nc.vec[m] <- mean(nc.data$Y)

  m1.cf.vec[m] <- mean(cf.data$M1)
  m2.cf.vec[m] <- mean(cf.data$M2)
  y.cf.vec[m] <- mean(cf.data$Y)

  y.med.vec[m] <- mean(mediation$Y)

  effects.y[m,1] <- y.cf.vec[m]-y.nc.vec[m] #TE
  effects.y[m,2] <- y.med.vec[m]-y.nc.vec[m] #TDE
  effects.y[m,3] <- effects.y[m,1]-effects.y[m,2] #NIE
  effects.y[m,4] <- effects.y[m,3]/effects.y[m,1] #% med
```

```
  effects.m1[m] <- m1.cf.vec[m]-m1.nc.vec[m]
  effects.m2[m] <- m1.cf.vec[m]-m2.nc.vec[m]

  print(m)


}
```

Supplementary file 9: Distributions of mediators (as percentages) in the subgroups used in subgroup analyses

	SFH	PD	SA	VV	VC	OHP	NEET	GPA1	GPA2	GPA3	GPA4
Quintiles of childhood income							%				
Lowest	0.4	11.3	2.0	0.4	4.5	6.5	13.5	40.2	26.3	20.0	13.5
2nd	0.3	9.8	1.5	0.3	3.0	3.6	8.8	33.5	26.6	22.6	17.2
3rd	0.2	8.3	1.2	0.2	2.1	2.0	6.3	27.7	25.8	25.5	21.0
4th	0.2	7.4	1.0	0.2	1.7	1.3	4.8	21.8	23.8	27.6	26.9
Highest	0.2	7.0	0.9	0.2	1.1	0.8	4.3	13.9	19.0	28.9	38.2
Parental psychiatric disorder											
No	0.2	7.9	1.2	0.3	2.3	2.3	7.1	26.8	24.2	25.2	23.9
Yes	0.6	18.0	2.4	0.4	4.1	8.4	11.9	34.6	25.9	21.7	17.8
Parental substance abuse											
No	0.3	8.3	1.2	0.2	2.2	2.3	7.1	26.4	24.2	25.3	24.1
Yes	0.6	15.8	3.1	0.6	6.3	11.1	14.0	43.4	25.9	18.7	11.9
Parental violent crime											
No	0.3	8.4	1.2	0.2	2.2	2.4	7.2	26.6	24.2	25.3	23.9
Yes	0.6	15.9	3.3	0.7	8.1	11.5	15.7	44.9	26.1	17.9	11.0
Family stability											
Intact two-parent	0.2	6.9	1.0	0.2	1.8	1.4	5.9	23.9	23.8	26.3	26.1
Intact single-parent	0.5	13.9	2.4	0.4	4.5	6.8	12.8	37.3	25.5	21.1	16.2
Multiple changes	0.5	13.7	2.5	0.6	5.0	7.4	12.0	39.8	26.6	20.3	13.3
Ever without parents	0.6	23.7	3.0	0.8	7.2	19.7	20.8	44.2	22.3	17.9	15.6
Disrupted two-parent	0.4	12.7	1.9	0.4	3.7	5.4	10.3	34.7	26.0	22.1	17.2
Repartnered single-parent	0.4	13.0	2.4	0.5	4.5	5.6	11.2	37.5	26.3	21.6	14.7

SFH: Self-harm 13-17
PD: Psychiatric disorder 13-17
SA: Substance abuse 13-17
VV: Violent victimization 13-17
VC: Violent crime 13-17
OHP: Out-of-home placement 13-17
NEET: Not in Education, employment or training 13-17
GPA1-GPA4: Quartiles of grade point average
